# Core–Shell PLGA Nanoparticles: In Vitro Evaluation of System Integrity

**DOI:** 10.3390/biom14121601

**Published:** 2024-12-14

**Authors:** Tatyana Kovshova, Julia Malinovskaya, Julia Kotova, Marina Gorshkova, Lyudmila Vanchugova, Nadezhda Osipova, Pavel Melnikov, Veronika Vadekhina, Alexey Nikitin, Yulia Ermolenko, Svetlana Gelperina

**Affiliations:** 1 Faculty of Chemical and Pharmaceutical Technologies and Biomedical Preparations, D. Mendeleev University of Chemical Technology of Russia, Miusskaya pl. 9, Moscow 125047, Russia; j.malinowskaya@gmail.com (J.M.); juliakot1412@gmail.com (J.K.); kompacc@yandex.ru (N.O.); uve2007@yandex.ru (Y.E.); 2 Laboratory of Polyelectrolyte Chemistry and Biomedical Polymers, Topchiev Institute of Petrochemical Synthesis, Russian Academy of Sciences, Leninsky Prosp. 29, Moscow 119991, Russia; mgor@ips.ac.ru (M.G.); vanchugowa.lyudmila@yandex.ru (L.V.); 3 Department of Fundamental and Applied Neurobiology, V. Serbsky Federal Medical Research Centre of Psychiatry and Narcology of the Ministry of Health of the Russian Federation, Kropotkinskiy per. 23, Moscow 119034, Russia; proximopm@gmail.com (P.M.); veronikavadehina@gmail.com (V.V.); 4 Laboratory of Biomedical Nanomaterials, National University of Science and Technology (MISIS), Leninsky Prosp. 4, Moscow 119049, Russia; nikitin.chemistry@mail.ru

**Keywords:** core–shell nanoparticles, PLGA, HSA, DIVEMA, poloxamer 188, Gl261 cells, confocal microscopy

## Abstract

The objective of this study was to compare the properties of core–shell nanoparticles with a PLGA core and shells composed of different types of polymers, focusing on their structural integrity. The core PLGA nanoparticles were prepared either through a high-pressure homogenization–solvent evaporation technique or nanoprecipitation, using poloxamer 188 (P188), a copolymer of divinyl ether with maleic anhydride (DIVEMA), and human serum albumin (HSA) as the shell-forming polymers. The shells were formed through adsorption, interfacial embedding, or conjugation. For dual fluorescent labeling, the core- and shell-forming polymers were conjugated with Cyanine5, Cyanine3, and rhodamine B. The nanoparticles had negative zeta potentials and sizes ranging from 100 to 250 nm (measured using DLS) depending on the shell structure and preparation technique. The core–shell structure was confirmed using TEM and fluorescence spectroscopy, with the appearance of FRET phenomena due to the donor–acceptor properties of the labels. All of the shells enhanced the cellular uptake of the nanoparticles in Gl261 murine glioma cells. The integrity of the core–shell structures upon their incubation with the cells was evidenced by intracellular colocalization of the fluorescent labels according to the Manders’ colocalization coefficients. This comprehensive approach may be useful for the selection of the optimal preparation method even at the early stages of the core–shell nanoparticle development.

## 1. Introduction

Drug delivery by nanoparticles may considerably improve the safety and selectivity of many drugs by altering their pharmacokinetics and tissue distribution using endogenous mechanisms. The first success of this strategy was achieved in antibiotic therapy by exploiting the known ability of colloidal particles, especially hydrophobic ones, to accumulate in the resident macrophages of macrophage-rich organs, such as the liver, spleen, lungs, etc. (organs of the mononuclear phagocytic system (MPS)) [1]. Loading of antibiotics into the nanoparticles improved their penetration into macrophages, which serve as a niche for pathogens, and thus considerably enhanced their efficacy [2]. Later, the discovery of the enhanced permeability and retention (EPR) effect, which enables the extravasation and retention of colloids in tumors due to the differences between the vascular systems of solid tumors and normal tissues, inspired enormous interest in nanomedicines for cancer chemotherapy. Indeed, preclinical studies demonstrated numerous instances of the improved antitumor effect of nanoparticle-bound drugs due to their enhanced trafficking to tumors. However, it was soon realized that the accumulation of nanoparticles in the MPS organs, although it is useful for drug delivery to these organs, considerably interferes with their ability to target other sites in the body, decreasing their blood circulation half-life and targeting capability [3]. Moreover, the large heterogeneity of the EPR effect observed on both the intra- and interindividual levels leads to heterogeneous therapeutic outcomes [4,5,6]. Indeed, the advantage of the approved nanoformulations of classical anticancer drugs designed to exploit the EPR effect, such as Caelyx^®^, Abraxane^®^, Genexol-PM^®^, or Onivyde^®^, was achieved more due to their enhanced safety than due to any improvement in antitumor effect or patient survival [6].

These disappointing results have given rise to numerous new trends in pharmaceutical nanotechnology, starting from the use of theranostics for more insightful pre-selection of patients who could benefit from nanomedicines [7] to the development of delivery systems for the next generations of drugs (immune checkpoint inhibitors [8], nanoadjuvants [9], RNA-based therapeutics [10], drug combinations, and new types of nanocarriers [11,12]).

Among the latter, core–shell nanoparticles draw considerable attention; over the last few decades, they have been extensively studied for many applications, including in the biomedical field (as reviewed in [13,14,15,16,17]). This technology indeed enables the optimization of plain “first-generation” nanoparticles by improving their in vitro and in vivo stability, as well as offering the possibility to load them with both hydrophilic and hydrophobic agents and tune their release profiles. Moreover, hydrophilic shells may shield nanoparticles from macrophages (the stealth effect), thus optimizing their biodistribution profile and enhancing their capability to reach other organs besides the MPS. Shells rich in functional groups could also enhance the feasibility of nanoparticle surface modification and conjugation with drugs or ligands for targeted delivery.

Biodegradable and biocompatible poly(lactide-co-glycolic) acid (PLGA) nanoparticles, some of the most popular nanocarriers in drug delivery [18], appear to be good candidates for this approach. These nanoparticles are relatively hydrophobic, and therefore their inherent disadvantage is their massive accumulation in macrophage-rich organs. Moreover, the attachment of bioligands that could enable active tumor targeting is difficult because the reactive groups on the surface of the PLGA nanoparticles are few (limited to the end carboxylic groups of the polymer) and sterically hindered.

To address these issues, various surface modification strategies have been developed to reduce opsonization and improve the pharmacokinetics and targeting efficiency of PLGA nanoparticles. The advantages of core–shell PLGA nanocarriers, such as steric stabilization, modulation of drug release profiles, and facilitated surface functionalization, have been demonstrated in numerous studies. Thus, PEGylation, which involves coating nanoparticles with polyethylene glycol (PEG), has been widely used to extend the nanoparticle circulation time by reducing opsonization. However, recent studies have raised concerns about the immune response triggered by PEG, including the production of anti-PEG antibodies, which can lead to accelerated clearance from the bloodstream and reduced therapeutic efficacy [19]. Other shell-forming agents include biopolymers such as chitosan, alginate, albumin, collagen, silk fibroin, etc. [13,20,21,22,23]. The methods used to create the shells primarily involve the adsorption of shell-forming polymers onto the nanoparticle surfaces, which can be performed either during their formation or by incubating preformed nanoparticles in a solution of the shell-forming agent [22]. Sometimes, cross-linking of the shell [24] or its conjugation with the PLGA core [25,26] is used to improve the system stability.

Clearly, the integrity of the core–shell structure is essential for the successful performance of the system in the biological environment. At the same time, this integrity has not always been well elucidated.

The objective of the present study was to evaluate the integrity of nanoparticles with a PLGA core and shells composed of different types of polymers in cell-free media and their integrity upon their interaction with cells, using Gl261 murine glioma cells as the model. The shell-forming polymers included the non-ionic polymeric surfactant poloxamer 188 (PLGA/P188 NPs), DIVEMA (a copolymer of divinyl ether with maleic anhydride, PLGA/DIVEMA NPs), and human serum albumin (PLGA/HSA NPs).

The methods of preparing the core–shell nanoparticles were developed in previous studies by us [27,28,29]. Due to their structure, the selected polymers create a hydrophilic shell on the nanoparticle surface and improve their colloidal stability. A considerable number of the functional groups exhibited by HSA (NH_2_, COOH, and SH) and DIVEMA (COOH) also facilitate the binding of ligands to the core–shell nanocarrier.

These polymers are well characterized and appear to be suitable shell-forming agents. Human serum albumin (HSA) has gained attention due to its non-immunogenic properties and its potential to enhance cellular uptake via the albumin receptors present on endothelial and tumor cells. This makes HSA a good alternative to PEG and a promising coating material for improving both the circulation time and targeting efficiency of PLGA NPs. Additionally, poloxamer 188, a non-ionic surfactant, has been shown to alter the biodistribution of polymeric nanoparticles, although it does not necessarily extend the circulation time. Thus, coating PLGA nanoparticles with poloxamer 188 altered their biodistribution and enabled brain delivery of encapsulated doxorubicin; importantly, this formulation also improved doxorubicin’s safety profile compared to that of the free drug [30]. Moreover, both poloxamer 188 and HSA are effective surfactants and stabilizers with a long history of application in pharmaceutical products [31,32].

Another promising approach is the modification of PLGA NPs with a polyanion, such as DIVEMA. This modification significantly hydrophilizes the nanoparticles’ surface, reducing opsonization and enhancing their stability in the bloodstream. Furthermore, the carboxyl groups introduced by DIVEMA offer additional opportunities for further functionalization, enabling the attachment of targeting ligands or other therapeutic agents. The relatively low toxicity of low-molecular-weight DIVEMA used in this study has been demonstrated in clinical studies [33]. A number of studies have revealed the broad spectrum of DIVEMA’s biological activities, such as its antiviral and antibacterial activity and induction of immune responses to tumors [34,35].

Apart from the physicochemical evaluation, the core–shell system integrity was monitored using fluorescent spectroscopy using the PLGA nanoparticles, the core and shell of which were labeled with pairs of fluorescent dyes with donor–acceptor properties (Cyanine5/Cyanine3 and Cyanine5/rhodamine B). These dye pairs are capable of exhibiting the Förster resonance energy transfer (FRET) phenomenon. This distance-dependent phenomenon enables the evaluation of core and shell integrity based on changes in the donor and acceptor fluorescence intensity, which are observed only when the molecules are localized in close proximity [36,37]. Internalization of the core–shell nanoparticles into Gl261 cells was investigated using confocal laser fluorescent microscopy.

## 2. Materials and Methods

### 2.1. Materials

PLGA (Purasorb^®^ PDLG 5004A, 50/50, η = 0.4 dL/g, acid terminated) was purchased from Corbion Biomaterials (Amsterdam, The Netherlands). N-(3-dimethylaminopropyl)-N′-ethylcarbodiimide hydrochloride (EDC), N,N′-dicyclohexylcarbodiimide (DCC), 4-(dimethylamino)pyridine (DMAP), N–hydroxysuccinimide (NHS), diisopropylethylamine (DIPEA), human serum albumin (HSA), polyvinyl alcohol (PVA, 9–10 kDa, 80% hydrolyzed), poloxamer 188 (P188), 2,2′-azobisisobutyronitrile (AIBN), rhodamine B (RhB), and rhodamine B isothiocyanate (RhBITC) were purchased from Sigma-Aldrich (St. Louis, MO, USA). The reactive derivatives of the fluorescent dyes Cyanine5 (Cy5) amine and Cyanine3 (Cy3) amine were purchased from Lumiprobe (Moscow, Russia). D-mannitol was purchased from Dia-M (Moscow, Russia). All the other chemicals were of analytical grade.

### 2.2. Preparation of Core–Shell PLGA Nanoparticles

#### 2.2.1. Preparation of Dual-Labeled Nanoparticles with a PLGA Core and an HSA Shell (PLGA-Cy5/HSA-RhBITC NPs)

For fluorescence labeling, conjugates of PLGA with Cyanine5 amine (PLGA-Cy5) and HSA with rhodamine B isothiocyanate (HSA-RhBITC) were synthesized. PLGA-Cy5 was synthesized by conjugating the PLGA’s terminal group with the water-soluble amine derivative of the Cyanine5 dye (Cy5; λ_ex_ = 651 nm; λ_em_ = 670 nm) using the NHS/EDC coupling reaction in the presence of DIPEA, as previously described in [27,38,39]. Briefly, solutions of Cy5 amine, DIPEA, EDC, NHS, and PLGA in dichloromethane (DCM) were combined, and the reaction mixture was stirred in the dark (for 48 h, at ambient temperature). The resulting solution was washed three times with water and a water/methanol (1:1) mixture. The organic phase was then dried over anhydrous sodium sulfate, and the solvent was removed through evaporation. The residue was dissolved in ethyl acetate, and the polymer was precipitated by adding hexane (at a 10-fold volume). The Cy5 content in the dried conjugate was measured spectrophotometrically. The PLGA-Cy5 conjugate was used in the experiments with a dye-to-polymer ratio of 1:600 (*w*/*w*).

The PLGA-Cy5 conjugate was analyzed by using permeation chromatography as described in [27]. Briefly, the analysis was performed using a Waters HPLC system equipped with a set of Styrogel HR5E and HR4E columns, a refractive index detector (Waters 2414 RI Detector, Waters Corporation, Milford, MA, USA), and a UV detector (Milton Roy UV detector model 3100, Citra, FL, USA, λ = 264 nm). Tetrahydrofuran was used as the solvent and the eluent at a flow rate of 1.00 mL/min. The data were analyzed using Z-lab software v1.

HSA was conjugated with rhodamine B isothiocyanate following the method described by Yang et al. [40]. Briefly, rhodamine B isothiocyanate was dissolved in DMSO, and this solution was added dropwise to a 1% HSA solution (0.15 mM) in a 9:1 *v*/*v* mixture of saline (0.15 M NaCl) and buffer (0.15 M NaHCO_3_, pH 9.0). The RhBITC/HSA molar ratio was 5:1. The solution obtained was stirred overnight under cooling. A NH_4_Cl solution (50 mM) was then added to stop the reaction, followed by an additional hour of stirring. The resulting fluorescently labeled HSA was purified using dialysis against phosphate-buffered saline (PBS, 0.01 M PBS, pH 7.4) at 4 °C in the dark. The concentration of RhBITC was determined by calculating the fluorophore/protein molar ratio (F/P):

(1) $$C\;\left(HSA-RhBITC\right)=\frac{A_{280\;}-CF\times A_{558}}{\varepsilon_{HSA}}\times dilution\;factor$$

(2) $$\frac{F}{P}=\frac{A_{558}}{\varepsilon_{RhBITC}\times C\;\left(HSA-RhBITC\right)\;}\times dilution\;factor$$

where A_558_ represents the absorbance (A) of RhBITC at 558 nm; A_280_ is the absorbance (A) of HSA-RhBITC at 280 nm; ε_HSA_ is the molar extinction coefficient of HSA; ε_RhBITC_ is the molar extinction coefficient of RhBITC; CF is the correction factor to account for the dye’s absorbance at 280 nm; and the dilution factor accounts for any dilution of the protein:dye sample for the absorbance measurements. The HSA-RhBITC conjugate used in further experiments had an F/P ratio of 1.25.

Core–shell nanoparticles were prepared using the high-pressure homogenization–solvent evaporation technique (o/w). The HSA shell was formed using three different techniques: by using HSA as a stabilizer during nanoparticle formation (interfacial embedding of HSA) or by adsorbing or conjugating HSA onto previously prepared PLGA nanoparticles without a shell (Supplementary Figure S1) [28].

Interfacial embedding of HSA: A solution of a 1:1 mixture of PLGA and PLGA-Cy5 (150 mg + 150 mg) in dichloromethane was emulsified with an aqueous 0.5% HSA-RhBITC solution (30 mL) using a high-shear homogenizer (Ultra-Turrax T18 Basic, IKA-Werke, GmbH, Staufen, Germany). This coarse emulsion was further homogenized at high pressure (15,000 psi, Microfluidizer L10, Microfluidics, Newton, MA, USA), and the solvent was removed through vacuum evaporation. The resulting suspension was then filtered through a sintered glass filter and freeze-dried using 2.5% (*w*/*v*) D-mannitol as a cryoprotectant. The freeze-dried nanoparticles were stored at 4 °C. Unbound HSA was removed through repeated washing and centrifugation of the nanosuspension (48,060× *g*, 20 °C, 20 min). A portion of the sample was subjected to shell cross-linking with glutaraldehyde to improve the shell stability [28]. Based on the findings of Langer et al. [41], 117 µL of an 8% glutaraldehyde solution (~0.1 mmol) per 1 mg of HSA (15 µmol) was added to the reaction mixture to ensure reliable HSA cross-linking.

Adsorption of HSA: To prepare PLGA/HSA nanoparticles through adsorption, PLGA-Cy5 nanoparticles were first prepared using 1% polyvinyl alcohol (PVA, 9–10 kDa, 80% hydrolyzed, Merck, Darmstadt, Germany) as a stabilizer, as described in [38]. Then, the nanoparticles were washed with water and resuspended in a 0.5% aqueous HSA-RhBITC solution, followed by 30 min of incubation under continuous stirring (37 °C, 130 rpm). Unbound HSA-RhBITC was removed through repeated centrifugation (48,060× *g*, 18 °C, 10 min). The supernatants were successively analyzed using spectrophotometry for the presence of unbound HSA. A portion of the sample was subjected to HSA cross-linking [28], followed by freeze-drying, as described above.

Conjugation of HSA: HSA amino groups were conjugated with the carboxyl end groups of PLGA through a coupling reaction using 1-ethyl-3-(3-dimethylaminopropyl)carbodiimide hydrochloride (EDC) in phosphate buffer (1 h, 0.05 M, pH 6.8) [42]. An indirect acid–base titration in MES buffer (0.05 M, pH 5) revealed that the PLGA-Cy5 nanoparticles contained 2.4 µmol-eq/mL of functional carboxyl groups. A 10-fold molar excess of EDC (24 µmol-eq/mL, 0.46 mg/mL NP) and NHS (10-fold excess: 24 µmol-eq/mL, 0.46 mg/mL NP) was then added to the nanoparticle suspension. The mixture was incubated at 37 °C for 2 h under stirring (90 rpm), washed through centrifugation (48,060× *g*, 30 min, 25 °C), and resuspended in MES buffer (0.05 M, pH 5). Activated nanoparticles were then incubated in a 1% HSA-RhBITC solution under continuous stirring (90 rpm, 1 h, 37 °C). Unconjugated HSA was removed through two-step washing (centrifugation at 48,060× *g*, 10 min, 18 °C). The precipitated nanoparticles were resuspended in HEPES buffer (0.1 M, pH 7.4). The HSA content in the supernatants was assessed spectrophotometrically based on the fluorescent properties of the labeled polymer. The nanosuspensions were then freeze-dried using 2.5% mannitol as a cryoprotectant.

#### 2.2.2. Preparation of Dual-Labeled Nanoparticles with a PLGA Core and a DIVEMA Shell (PLGA-Cy5/DIVEMA-Cy3 NPs)

The alternating copolymer of divinyl ether and maleic anhydride (DIVEMA) was synthesized through radical cyclopolymerization of the corresponding monomers in dry acetone, using 2,2′-azobisisobutyronitrile (AIBN) as the initiator and tetrahydrofuran as the chain transfer agent [27,43]. The polymer structure was confirmed through IR and NMR spectroscopy, as previously described in [27]. A sample of DIVEMA copolymer with a molecular weight (MW) of 23 kDa and a characteristic viscosity of 0.16 dL/g (measured in borate buffer containing 0.2 M NaCl) was used in the following experiments.

Fluorescent labeling of DIVEMA was achieved by conjugating the carboxyl groups with the water-soluble amine derivative of Cyanine3 dye (Cy3; λ_ex_ = 555 nm; λ_em_ = 570 nm) via a carbodiimide coupling reaction, as described in [27]. First, the DIVEMA carboxyl groups (2.26 mmol DIVEMA solution in acetone [600 mg in 30 mL of acetone]) were activated using NHS (23.4 mg, 0.20 mmol in 1 mL of acetone) for 30 min under continuous stirring in the dark. A dicyclohexylcarbodiimide (DCC) solution (14.1 mg [68.3 μmol] in 0.5 mL of acetone) and a Cy3 amine solution in a mixed organic solvent (18 mg [28.7 μmol] in 1 mL acetone and 0.5 mL ethanol) were then added. The mixture was incubated for 3 h under constant stirring, after which the conjugate was purified through three-fold precipitation in diethyl ether (ten times the volume). The precipitate was separated and washed with chloroform. The absence of unbound Cy3 in the wash solvent was monitored using UV spectroscopy at 543 nm. The product was dried under a vacuum to a constant weight. The resulting purified DIVEMA-Cy3 conjugate was stored in an evacuated flask or under an inert gas atmosphere. The conjugate formation was confirmed through gel permeation chromatography using a refractive index detector and a UV detector, as previously described [27]. The Cy3 content was quantified using UV spectroscopy at λ_max_ = 552 nm. Conjugate containing 2.7% *w*/*w* of the dye (at a dye-to-polymer ratio of 1:40, *w*/*w*) was used in the experiments.

Preparation of the PLGA-Cy5/DIVEMA-Cy3 nanoparticles was undertaken using a high-pressure homogenization–solvent evaporation technique (PLGA-DIVEMA-H): A 1:1 mixture of PLGA and the PLGA-Cy5 polymers (2 × 300 mg [2 × 2.3 mmol]) was dissolved in 7.2 mL of DCM. The resulting solution was mixed with a solution of DIVEMA-Cy3 in acetone (71.4 mg [0.268 mmol] in 4.8 mL), and the organic phase was added to a 0.5% aqueous PVA solution (60 mL). The mixture was first emulsified using a high-shear homogenizer (Ultra-Turrax T18IKA Industrie- und Kraftfahrzeugausrüstung GmbH, Königswinter, Germany, 23,600 rpm) and then subjected to high-pressure homogenization (Microfluidics M-110P, Microfluidics, Newton, MA, USA, 15,000 psi). Afterwards, the organic solvent was removed under a vacuum, and the resulting suspension was filtered through a sintered glass filter (pore size: 90–150 μm) and freeze-dried using 2.5% (*w*/*v*) of D-mannitol as a cryoprotectant. After lyophilization, the nanoparticles were resuspended in purified water and subjected to centrifugation: first at 10,000× *g* (10 min, 5 °C) to remove agglomerates and then at 48,060× *g* (two times, 30 min, 5 °C). The resulting nanosuspension was freeze-dried with 2.5% mannitol as a cryoprotectant and stored at 4 °C. Plain PLGA nanoparticles (PLGA without a shell) were obtained in a similar way, with the addition of 4.8 mL of acetone without DIVEMA to the polymer solution.

Preparation of PLGA-Cy5/DIVEMA-Cy3 nanoparticles through nanoprecipitation (PLGA-DIVEMA-N): The PLGA/PLGA-Cy5 (1:1) mixture (60 mg; 0.46 mmol) was dissolved in a mixture of acetonitrile and acetone (1:2 *v*/*v*). Then, 24.7 mg (93 μmol) of DIVEMA-Cy3 was added, and the resulting solution was added dropwise to 60 mL of dH_2_O under stirring (1000 rpm) and incubated for 4 h. The organic solvents were evaporated under a vacuum. The resulting suspension was filtered through a sintered glass filter (pore diameter: 90–150 μm) and freeze-dried with the addition of 2.5% (*w*/*v*) mannitol as a cryoprotectant. Plain PLGA nanoparticles (PLGA without a shell) were obtained similarly but without DIVEMA and using 0.5% aqueous PVA solution instead of purified water.

#### 2.2.3. Preparation of PLGA Nanoparticles Coated with Fluorescently Labeled Poloxamer 188

Synthesis of poloxamer 188 conjugate with RhB (P188-RhB). The conjugate of poloxamer 188 with rhodamine B (P188-RhB) was synthesized and characterized as reported previously [29]. Briefly, a solution of P188 (1000 mg, 0.12 mmol), RhB (126 mg, 0.26 mmol), EDC (55 mg, 0.29 mmol), and 4-(dimethylamino)pyridine (DMAP, 59 mg, 0.48 mmol) in dimethylformamide (DMF, 15 mL) was stirred at ambient temperature for 3 days in the dark. Afterwards, the mixture was diluted with Et_2_O, and the crude product was precipitated overnight in a freezer at −20 °C. The precipitate was washed three times with a mixture of DMF/Et_2_O (1:1) while cooling. The crude product was then purified through gel filtration using a Sephadex G25 column. The combined fractions containing the product were then lyophilized. The absence of unbound dye was confirmed using thin-layer chromatography using a mixture of *i*-PrOH/H_2_O (3:5) as the eluent. The stability of the conjugate in PBS (pH 7.4), DMEM, or human plasma was also evaluated using TLC (incubation for 2 h at 37 °C). Conjugate containing 4.49% *w*/*w* of the dye was used in the experiments.

Evaluation of poloxamer 188’s adsorption onto the PLGA nanoparticles’ surface: The PLGA-Cy5-P188-RhB nanoparticles were prepared by incubating the PLGA-Cy5 NPs (prepared using the high-pressure homogenization—solvent evaporation technique as described above) in a 1% aqueous solution of P188-RhB for 30 min.

The evaluation of P188-RhB’s adsorption was carried out as reported previously [29]. To analyze the P188-RhB content in one vial, the nanoparticle lyophilizate was resuspended in 1.5 mL of the 1% P188-RhB solution. The suspension was incubated at room temperature under stirring at 130 rpm. The aliquots (400 μL) collected after 30, 90, and 180 min were centrifuged to separate the PLGA NPs (48,068× *g*, 30 min, 5 °C). The precipitated nanoparticles were washed with water (1 mL), dried, and dissolved in DMSO (2 mL). The P188-RhB content in the resulting solution was determined spectrophotometrically at λ_max_ = 555 nm (using a UV-1900i spectrophotometer, Shimadzu Corp, Kyoto, Japan) using the calibration curve. The amount of P188-RhB in mg/m^2^ adsorbed onto the PLGA NPs’ surface was calculated as follows:

(3) $$S=\frac{cV_{s}\rho_{PLGA}d}{6m},$$

where m is the mass of PLGA nanoparticles in a vial, mg; c is the concentration of the P188 RhB conjugate in the analyzed sample, determined from the calibration curve, mg/mL; V_s_ is the sample volume, 2 mL; ρ_PLGA_ is the PLGA’s density, 1.2 g/cm^3^; and d is the nanoparticles’ diameter, nm.

### 2.3. Characterization of the Nanoparticles

The mean hydrodynamic size and polydispersity index (PDI) of the nanoparticles were measured using dynamic light scattering (DLS) using a Zetasizer NanoZS (5 mW He-Ne laser; operating wavelength: 633 nm; Malvern Instruments, Malvern, UK). The zeta potential was determined using electrophoretic light scattering (ELS) in a U-shaped disposable folded capillary cell using the same instrument. Measurements of all the samples were performed in triplicate. The samples were diluted to a concentration of 0.2 mg/mL polymer in Milli-Q water.

The total PLGA concentration in all of the samples was quantified using capillary electrophoresis (CAPEL 105M, Lumex, St. Petersburg, Russia), as described previously [44]. The amount of PLGA was assessed according to the amount of lactate formed after nanoparticle hydrolysis in 1 M NaOH (37 °C, 24 h, continuous shaking at 200 rpm). The content of the –COOH groups in the PLGA nanoparticles was determined through potentiometric titration [44].

The dye content in the nanoparticles was quantified using UV spectroscopy (Shimadzu UV-1800, Kyoto, Japan). The Cy3 and Cy5 contents in the nanoparticles were measured at 553 nm and 649 nm for Cy3 and Cy5, respectively, after dissolving the core–shell nanoparticles in DMSO. The HSA-RhBITC shell content in the nanoparticles was calculated indirectly according to the difference between the content of HSA-RhBITC (determined using UV spectroscopy at λ_max_ = 558 nm in water) added and washed in the process of nanoparticle preparation as follows:

(4) $${m(HSA)}_{NP}={\;m(HSA)}_{added}-{m(HSA)}_{washed},$$

where m(HSA)_NP_ is the content of HSA-RhBITC on the nanoparticles’ surface; m(HSA)_added_ is the content of HSA-RhBITC added during nanoparticle synthesis; and m(HSA)_washed_ is the content of HSA-RhBITC washed during nanoparticle synthesis.

Fourier transform infrared (FTIR) spectroscopy of the polymer–dye conjugates and the reference samples was carried out using a PerkinElmer Spectrum One FTIR spectrometer equipped with universal ATR sampling accessories with diamond/ZnSe crystals (PerkinElmer Inc., Shelton, CT, USA).

The morphology of the core–shell nanoparticles was studied through transmission electron microscopy (TEM) using a JEOL JEM-1400 electron microscope (JEOL, Tokyo, Japan) at an accelerating voltage of 120 kV. A diluted aqueous nanosuspension was applied to Cu grids coated with a Formvar polymer film, and the solvent was allowed to dry completely. To improve the image quality, additional negative contrasting of the nanoparticles was performed using the UranyLess EM stain (Electron Microscopy Sciences, Hatfield, PA, USA).

### 2.4. Evaluation of Fluorescent Properties

The quantum yield and brightness of the fluorescently labeled nanoparticles were assessed as reported previously [39]. The light absorption values and the integrated fluorescence intensities were determined for each nanoparticle type.

The quantum yields of the nanoparticles were calculated as follows:

(5) $$\varphi =\varphi_{st}\frac{tg\;\alpha }{tg\;\alpha_{st}}{\left(\frac{n}{n_{st}}\right)}^{2},$$

where φ and φ_st_ are the quantum yield of the dye-labeled nanoparticles and the standard, respectively; tg α and tg α_st_ are tangents of the slope of the dependences of the integrated fluorescence intensity on light absorption for the test compound and the standard, respectively; and n and n_st_ are the refractive indices of the media in which the measurements were made.

The brightness of the nanoparticles was calculated as follows [45]:

(6) B=φ·ε·N,

where ε is the extinction coefficient of the dye determined via Beer–Lambert law, L∙mol^−1^∙cm^−1^; φ is the quantum yield; and N is the number of fluorescent dye molecules encapsulated inside the nanoparticles.

RhB (φ = 0.7, ε = 106,000 M^−1^ cm^−1^ [46]) was used as a reference to determine the quantum yield and brightness of Cy3-, RhB-, or RhBITC-labeled core–shell nanoparticles. Cy5 (φ = 0.2, ε  = 250,000 M^−1^ cm^−1^ [47]) was used as a reference to determine the quantum yield and brightness of the Cy5-labeled nanoparticles.

Fluorescence spectroscopy FRET studies: The fluorescence spectra were registered for nanoparticles with either one or two fluorescent labels and free dyes. The following types of nanoparticles were used for the study: PLGA-Cy5/HSA NPs, PLGA/HSA-RhBITC NPs, PLGA-Cy5/HSA-RhBITC NPs, PLGA/DIVEMA-Cy3 NPs, PLGA-Cy5/DIVEMA-Cy3 NPs, PLGA-Cy5/P188 NPs, PLGA/P188-RhB NPs, and PLGA-Cy5/P188-RhB. Nanoparticle suspensions were prepared in water so that the fluorescent label content in the nanoparticles with one label was equal to the content of the same dye in the nanoparticles with two fluorescent labels. The fluorescence spectra of the PLGA/HSA NPs and PLGA/P188 NPs were recorded at λ_ex_ = 550 nm over the emission range of λ_em_ = 565–800 nm and at λ_ex_ = 644 nm over the range of λ_em_ = 655–800. The fluorescence spectra for the PLGA/DIVEMA NPs were taken at λ_ex_ = 530 nm over the emission range of λ_em_ = 545–740 nm and at λ_ex_ = 630 nm over of the range of λ_em_ = 640–800 nm.

### 2.5. Evaluation of Core–Shell Nanoparticles’ Stability Using Physicochemical Methods

The colloidal stability of the nanoparticles was assessed at different time points by measuring their hydrodynamic diameters (using DLS) at 37 °C in simulated physiological media: PBS (pH 7.4; 0.15 M) and a 4.5% HSA solution in PBS.

The stability of the core–shell structures was also evaluated by assessing the amount of fluorescently labeled shell remaining on the nanoparticles’ surface through fluorescence measurements. The nanosuspensions (100 μg/mL as PLGA) were incubated in PBS (pH 7.4; 0.15 M), PBS (pH 7.4; 0.15 M) containing 4.5% HSA, or PBS (pH 5.5; 0.15 M) containing 4.5% HSA. At selected time points (0, 0.5, 1, 2, 4, and 6 h), 400 μL aliquots were taken, and then the fluorescence spectra of the suspensions were recorded. Then, the aliquots were centrifuged (48,060× *g*, 18 °C, 20 min) to precipitate the nanoparticles, and the fluorescence intensity of each supernatant was measured. The fluorescence spectra were recorded for HSA-RhBITC and P188-RhB (λ_ex_ = 560 nm; λ_em_ = 570–800 nm), PLGA-Cy5 (λ_ex_ = 649 nm; λ_em_ = 660–800 nm), and DIVEMA-Cy3 (λ_ex_ = 530 nm; λ_em_ = 540–800 nm). The percentage of the detached shell at each time point was calculated as the ratio of the fluorescence intensities in the supernatant to those in the nanosuspension.

### 2.6. Cells

Gl261 murine glioblastoma cells were purchased from the American Type Culture Collection (ATCC, Manassas, VA, USA). The cells were cultivated in DMEM supplemented with GlutaMax™ (2 mM, Gibco™, Thermo Fisher Scientific Inc., Bremen, Germany), 10% fetal bovine serum (Biowest, Lakewood Ranch, FL, USA) and penicillin (100 U/mL), and streptomycin (100 g/mL) (Gibco™). The cells were cultured under the standard conditions (37° C, 5% CO_2_) and used between passages 5 and 9.

### 2.7. The Cell Viability Assay

The effect of the core–shell nanoparticles on Gl261 cell viability was evaluated using the MTS assay (CellTiter 96 AQueous One Solution, PROMEGA, Madison, WI, USA), according to the manufacturer’s protocol (TB245). The Gl261 cells were cultured in 96-well plates (approximately 4000 cells per well, Corning, New York, NY, USA) in DMEM supplemented with 10% FBS for 48 h prior to the experiment. The culture medium was then removed and replaced with 100 µL of the nanoparticle suspensions at concentrations ranging from 0.625 to 100 µg/mL (PLGA concentration). Cells treated with the culture medium alone served as the negative control. After 48 h of incubation, the medium was replaced with fresh culture medium, and 20 µL of the MTS reagent was added to each well. The absorbance was measured at 490 nm following 3 h of incubation at 37 °C. Optical density was recorded using a Perkin Elmer EnSpire 2300 MultiMode Microplate Reader (Perkin Elmer, Shelton, CT, USA). Cell viability was calculated as the percentage ratio of the absorbance of the sample well to that of the control well and expressed as the relative cell viability (%). All of the tests were performed in triplicate.

### 2.8. Investigation of Nanoparticle Internalization Using Confocal Microscopy

Images were obtained using a Nikon A1R MP+ multiphoton confocal microscope (Nikon Instr., Inc., New York, NY, USA) equipped with four lasers—405 nm (λ_em_ = 425–475 nm), 488 nm (λ_em_ = 500–550 nm), 561 nm (λ_em_ = 570–620 nm), and 638 nm (λ_em_ = 663–738 nm)—and Plan Apo 20x/0.75 DIC N, Apo IR 60x/1.27 WI, and Apo TIRF 60x/1.49 oil DIC objectives. The data were analyzed using NIS-Elements AR Software v5.21.02. Gl261 cells were seeded onto confocal 35 mm high coverslip dishes (Ibidi, Fitchburg, WI, USA) (70 × 10^3^ cells) 48 h prior to the study. The cells were incubated with the samples for 15, 30, and 45 min (final concentration = 100 μg/mL in serum-containing medium). The cells were then washed three times with DPBS to remove the nanoparticles from the cell surface. Lysosomes were stained using LysoTracker Green DND26 (50 nM, Molecular Probes, Eugene, OR, USA). Plates were transferred to the microscope stage for live-cell imaging.

To assess the core–shell structure integrity of the nanoparticles as well as their colocalization with lysosomes (for the evaluation of their major NP internalization pathway and intracellular localization), the Manders’ overlap coefficient (MOC) values were calculated. The MOC values were calculated within the selected ROIs—with three random ROIs per image. Colocalization plots were built to compare the core–shell integrity of the nanoparticles (HSA, DIVEMA, or P188 shells with PLGA cores) obtained using different techniques.

### 2.9. Investigation of Nanoparticle Uptake Using Flow Cytometry

The Gl261 cells were seeded into 24-well plates (Corning) (100 × 10^3^ cells/well) and incubated for 24 h to allow for attachment and the obtainment of 70–90% confluence. Then, the samples were added to a final concentration of 100 μg/mL into the cell culture medium (DMEM supplemented with 10% FBS) and incubated for 15, 30, and 45 min.

At the specified time points, the cells were washed twice with HBSS buffer, detached using TrypLE solution (ThermoFisher, Waltham, MA, USA), and centrifuged at 1000× *g* (5 min, 18 °C). The cell pellets were resuspended in FACS buffer and analyzed on the FACS MoFlo XDP (Beckman Coulter, Brea, CA, USA). The nanoparticle uptake by the cells was determined as the percentage of NP-positive cells: PLGA-Cy5-positive or double-positive (PLGA-Cy5- and HSA-RhBITC//DIVEMA-Cy3/RhB-positive cells).

### 2.10. Statistics

The data were analyzed using GraphPad Prism 9 software (San Diego, CA, USA) with one- or two-way ANOVA followed by Tukey’s and Dunnett’s multiple comparisons tests. Characterization of the particle size, size distribution, and zeta potential and all of the in vitro measurements were conducted in triplicate.

## 3. Results

### 3.1. Core–Shell Nanoparticle Preparation and Characterization

The core PLGA nanoparticles were prepared through the commonly used method of high-pressure homogenization followed by solvent removal. This method involves the emulsification of a PLGA solution in DCM in an aqueous solution of polyvinyl alcohol (PVA), one of the most popular stabilizers used for the preparation of PLGA nanoparticles [31]. The core–shell nanoparticles were prepared using previously established techniques, including the adsorption of the shell-forming polymers onto the previously prepared core PLGA nanoparticles (P188, HSA); interfacial embedding, achieved by adding the shell-forming polymer in the course of nanoparticle preparation (DIVEMA, HSA); or covalent linking of the shell to the PLGA’s carboxyl end groups (HSA) [27,28,29].

To enable visualization of the core–shell nanoparticles’ interaction with the cells, both the core- and shell-forming polymers were labeled with covalently bound bright fluorescent dyes. As shown in our previous studies, only covalent linking ensures stable retention of the fluorescent labels by nanoparticles, which helps to avoid the presence of artifacts in imaging the nanoparticles’ interactions with biological objects [39]. Therefore, the following dye–polymer conjugates were synthesized using carbodiimide chemistry: PLGA–Cyanine5 (PLGA-Cy5), HSA–rhodamine B (using an RhBITC derivative, HSA-RhBITC), poloxamer 188–rhodamine B (P188-RhB), and DIVEMA–Cyanine3 (DIVEMA-Cy3). The covalent binding of the dyes to the polymers was confirmed through GPC or TLC analysis, as described in [27,28,29].

Additionally, the conjugates were analyzed using FTIR spectroscopy (Supplementary Figures S1–S4). In the DIVEMA-Cy3 spectrum, the formation of the amide bond as a result of the condensation of the amine groups of the dye with the acid groups of DIVEMA was evidenced by the presence of an amide II band at 1560 cm^−1^ in the conjugate’s FTIR spectrum, corresponding to the structural fragment C-N-H (Supplementary Figure S1). This band was clearly visible in the differential spectrum, which also contained bands associated with the dye: 2928 cm^−1^—stretching vibrations of the CH groups from aliphatic chains; 1450 cm^−1^—stretching vibrations of the C-N bond; and 760 cm^−1^—stretching vibrations of N-H bonds. Binding of rhodamine B to P188 was previously confirmed by the characteristic absorption band of C=O bonds at 1721 cm^–1^, indicating the formation of the ester bond resulting from the condensation of the carboxylic groups of the dye with the hydroxyl groups of P188 (FTIR recorded in KBr tablets) [29]. The FTIR spectra of the other conjugates provided no significant insights.

**Figure 1:**
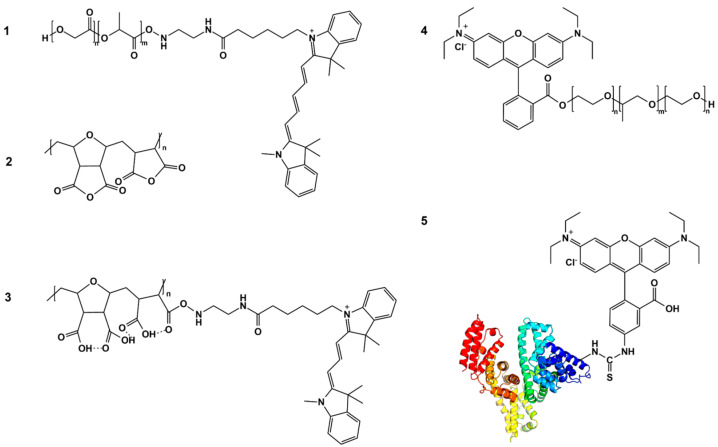
Chemical structures of fluorescently labeled polymers used for the preparation of core–shell nanoparticles: (**1**) PLGA conjugate with Cyanine5 (PLGA-Cy5), (**2**) DIVEMA in anhydride form, (**3**) DIVEMA conjugate with Cyanine3 (DIVEMA-Cy3), (**4**) poloxamer 188 conjugate with rhodamine B (P188-RhB), and (**5**) HSA conjugate with rhodamine B (HSA-RhBITC).

The chemical structures of the fluorescently labeled polymers are presented schematically in Figure 1.

**Figure 2:**
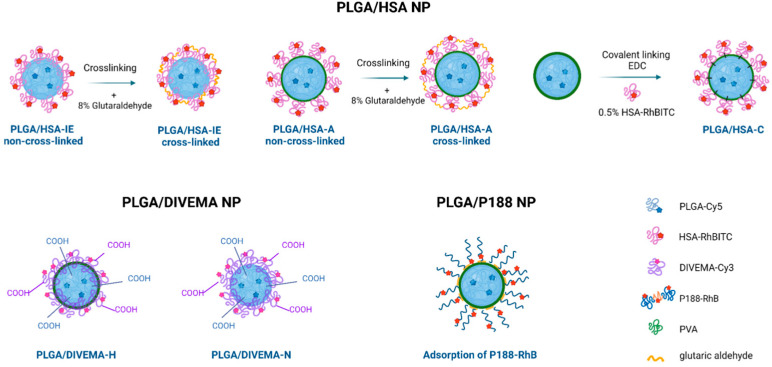
Schematic representation of the types of dual-labeled core–shell PLGA nanoparticles.

The types of dual-labeled core–shell PLGA nanoparticles prepared and investigated in this study are shown schematically in Figure 2 and Supplementary Figure S5. All of the nanoparticles appeared as stable opalescent suspensions (Supplementary Figure S6). The physicochemical parameters of these nanoparticles are shown in Table 1. The nanoparticles had hydrodynamic diameters of 100–250 nm (volume distribution) depending on the preparation method and shell type, and they were negatively charged—parameters considered optimal for drug delivery [48].

As seen from Table 1, the parameters of the PLGA/HSA nanoparticles depended on the method of shell formation. Thus, the adsorption and conjugation of HSA led to an increase in the hydrodynamic diameter (from ≈110 nm to ≈140 nm) and partial compensation for the negative surface zeta potential (from ≈−20 to ≈−7 mV) compared to the bare PLGA NPs, which correlated with the results of other authors [42]. In contrast, the interfacial embedding of HSA (acting as a surfactant in the absence of PVA) led to the formation of 90–100 nm particles with a higher negative zeta potential (≈ −30 mV). These data correlate with the results of Hyun et al., who observed similar tendencies for PLGA/HSA nanoparticles obtained through interfacial embedding and the physical adsorption of HSA [49]. It is noteworthy that cross-linking of the HSA shell did not exert any influence on the nanoparticles’ parameters compared to those of the non-cross-linked nanoparticles. The nanoparticles prepared using interfacial embedding with cross-linking (PLGA/HSA-IE cross-linked NPs) had a superior HSA/PLGA ratio (0.52 mg/mg PLGA, Table 1) compared to that of those prepared with the adsorption and conjugation methods. As suggested by Hyun et al. [49], the HSA introduced onto the nanoparticles’ surface during the emulsification stage could form a shell consisting of several HSA layers. The high density of HSA on the PLGA/HSA-IE NPs’ surface is also consistent with the relatively high negative surface charge, similar to the nanoparticles obtained in [42,49].

The fluorescently labeled PLGA-Cy5/DIVEMA-Cy3 NPs were prepared through either the high-pressure homogenization–solvent evaporation technique (PLGA/DIVEMA-H NPs) or nanoprecipitation (PLGA/DIVEMA-N NPs) (Figure 2). Dual labeling was achieved by using a 1:1 mixture of PLGA and the PLGA-Cy5 conjugate as the PLGA constituent and the DIVEMA-Cy3 conjugate. In both cases, the presence of DIVEMA led to a considerable increase in the mean hydrodynamic diameter (measured by DLS) (Table 2), which suggested the presence of a more pronounced hydration shell and abundant polyanionic groups on the surface of the hybrid nanoparticles. The sizes of the PLGA/DIVEMA-N and PLGA/DIVEMA-H NPs were about 180 nm and 264 nm, compared to 130 nm and 178 nm for the bare particles, respectively (Table 1). The content of the –COOH groups in both types of PLGA/DIVEMA NPs was considerably higher compared to that in the plain PLGA NPs: 0.72 ± 0.06 vs. 0.09 ± 0.04 mmol/g PLGA and 0.46 ± 0.05 vs. 0.04 ± 0.01 mmol/g PLGA for the PLGA/DIVEMA-H and PLGA/DIVEMA-N nanoparticles, respectively. Accordingly, these nanoparticles had higher negative zeta potentials compared to that of the plain PLGA NPs (Table 2). The relative content of the shell (DIVEMA-Cy3) was higher in the case of the nanoprecipitation method, which was probably due to the particle formation in the absence of PVA in the aqueous phase: 0.47 vs. 0.10 mg/mg PLGA, respectively (Table 1). Due to this more considerable shell, combined with their more favorable size and size distribution parameters, only the PLGA/DIVEMA-N NPs were used in further experiments.

The PLGA-Cy5/P188-RhB nanoparticles were prepared by incubating the plain nanoparticles in a 1% P188-RhB solution [29]. Conjugation of P188 with rhodamine B did not affect its surfactant properties: the amount of P188-RhB adsorbed onto the nanoparticles’ surfaces was even increased compared to that for P188 (0.38 vs. 0.14 mg/m^2^ of NP or 0.017 vs. 0.006 mg/mg PLGA, respectively). Modification of the PLGA NPs with poloxamer 188 and P188-RhB did not induce any changes in their hydrodynamic diameter or surface potential (Table 1), which correlated with our previous observations [50]. It is noteworthy that the P188 shell content was the lowest compared to that of the HSA-RhBITC and DIVEMA-Cy3 shells (0.017 mg/mg PLGA).

**Figure 3:**
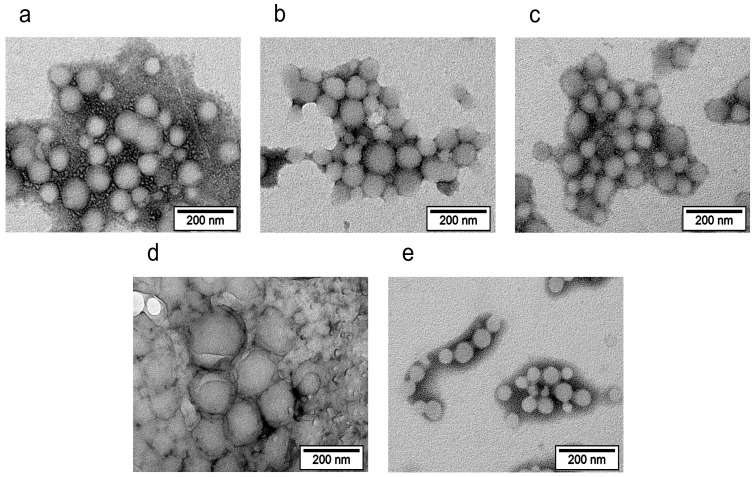
TEM images: (**a**) PLGA/HSA-C; (**b**) PLGA/HSA-A cross-linked; (**c**) PLGA/HSA-IE cross-linked; (**d**) PLGA/DIVEMA-N; (**e**) PLGA without a shell (JEOL JEM-1400 electron microscope, negative staining with UranyLess stain).

As shown using TEM, all types of the core–shell nanoparticles had a spherical shape (Figure 3). Although the shells of the PLGA/HSA NPs were not visualized, the presence of the hydrated HSA shell was confirmed by the smaller nanoparticle size observed using TEM compared to that from the DLS measurements of the same sample: 50–100 nm vs. 80–150 nm (Vol, PDI < 0.20). This observation correlates with the data obtained by other authors [51,52]. In the case of the PLGA/DIVEMA-N NPs, direct comparison of the sizes measured using TEM and DLS was not possible due to the high polydispersity of the PLGA/DIVEMA-N NPs (PDI > 0.2); however, it appears that in the TEM micrographs, the majority of the hybrid nanoparticles had smaller diameters than the diameters measured using DLS: ≈200 nm vs. 280 nm (Volume, Table 1). The sizes of the P188-RhB-coated nanoparticles observed using TEM were similar to those from the DLS measurements, which correlates with the low content of this shell compared to that in the other nanoparticle types.

### 3.2. Evaluation of Integrity Using Physicochemical Methods

**Figure 4:**
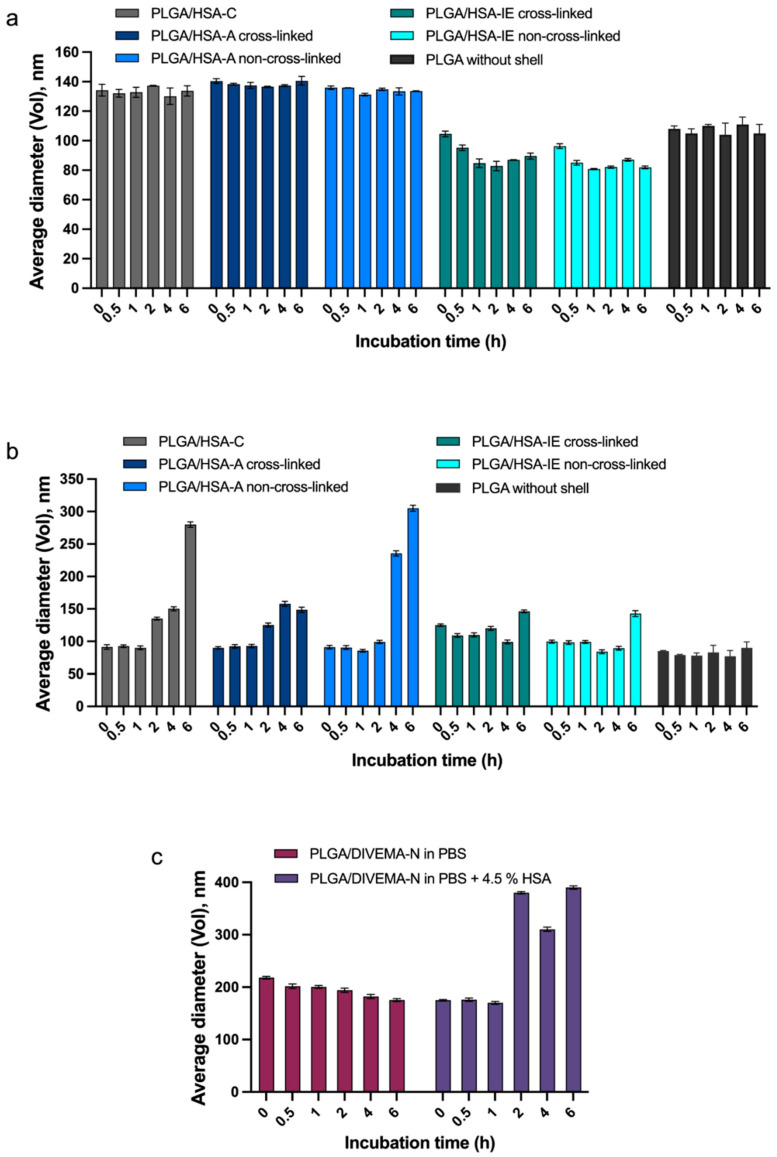
Average diameters of core–shell nanoparticles during 6 h of incubation in model media (pH 7.4; 37 °C; mean ± SD; n = 3): (**a**) PLGA-Cy5/HSA-RhB incubation in PBS; (**b**) PLGA-Cy5/HSA-RhB incubation in PBS in the presence of 4.5% HSA; and (**c**) PLGA-Cy5/DIVEMA-Cy3 incubation in PBS in the presence or absence of 4.5% HSA. PLGA nanoparticles without a shell were used as a control. Size measurements were performed using DLS (volume distribution).

The stability of the core–shell structures in biorelevant media was initially studied using DLS and spectrofluorimetry. As shown by the DLS measurements, all of the HSA- and DIVEMA-coated nanoparticles preserved their initial hydrodynamic diameters and polydispersity for at least 1 h (Supplementary Table S1) in the HSA-containing medium (pH 7.4, PBS buffer with 4.5% HSA) and for 6 h (Supplementary Table S2) in the absence of HSA (PBS, pH 7.4) (Figure 4).

When comparing the particle sizes using TEM (Figure 3) and the hydrodynamic diameter of the PLGA particles in the aqueous medium (Figure 4a), a significant contribution of the hydration shell to the hydrodynamic diameters of the particles is noted. According to Figure 4, the size of the PLGA nanoparticles without the shells, as measured using DLS in the presence of HSA, was lower (80–90 nm by volume) than that measured in water (100–110 nm). This may have been due to protein adsorption onto the surface of the nanoparticles and a change in the hydration shell’s size due to the compensation for the carboxyl group potential by the protein molecules. In the HSA medium, processes such as both the desorption of the shell and protein adsorption from the medium onto the surface of the particles can occur, which affects the hydration shell’s size and the calculated hydrodynamic diameter.

The next step involved evaluating the core–shell structure’s integrity in the nanoparticles using fluorescence spectroscopy based on the ability of the fluorescent dyes with donor–acceptor properties to exhibit the distance-dependent Förster resonance energy transfer (FRET) phenomenon. Thus, the cores and shells of the nanoparticles were labeled with pairs of fluorescent dyes, where Cy5 was the acceptor molecule and Cy3 and rhodamine B were the donors [53]. Normalized spectra of selected FRET pairs with Cy5 as the acceptor are shown in Supplementary Figure S7. Analysis of the absorption and emission spectra of the dyes in free form in ethanol demonstrated a significant overlap between the emission spectra of Cy3, RhB, and RhBITC and the absorption spectrum of Cy5. As a result, FRET (Förster resonance energy transfer) can be expected for these dye pairs, provided that the donor fluorophores in the labeled shell are in close proximity to the fluorophores of Cy5 attached to the polymer “core”.

As shown by the fluorescence measurements (Table 2), all of the dual-labeled PLGA nanoparticles had sufficient brightness for visualization based on a comparison of the parameters of the free dyes and nanoparticles (Supplementary Table S3) [46,47,54].

**Figure 5:**
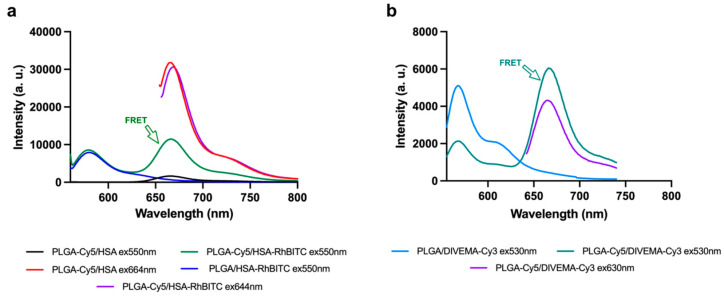
Fluorescence spectra of representative nanoparticle samples: (**a**) PLGA-Cy5/HSA-RhBITC (PLGA/HSA-IE cross-linked); (**b**) PLGA-Cy5/DIVEMA-Cy3 (PLGA/DIVEMA-N).

In the present study, FRET was detected for the Cy5—Cy3 (PLGA-Cy5/DIVEMA-Cy3 NP) and Cy5—RhBITC (PLGA-Cy5/HSA-RhBITC) pairs. As shown in Figure 5 (representative images), the fluorescence intensity of the acceptor in the double-labeled nanoparticles is detected when the excitation occurs at the donor’s wavelength (550 nm for RhBITC and 530 nm for Cy3). Normalized spectra of potential FRET pairs using Cy5 as the acceptor are shown in Supplementary Figure S7.

The FRET phenomenon was observed for all types of PLGA/HSA NPs, indicating the closeness of the shells and the PLGA cores (interaction between RhBITC and Cy5). Moreover, the most prominent FRET phenomenon was observed for the HSA shells obtained using the interphase embedding method (Supplementary Figure S8). Similarly, the PLGA/DIVEMA NPs demonstrated core–shell integrity through a strong FRET signal between Cy3 (DIVEMA) and Cy5 (PLGA), indicating the close proximity of the donor (in the PLGA core) and the acceptor (in the shell) (Figure 5b). No FRET phenomenon was detected for the PLGA/P188 NPs, which may have been due to the large distance between the donor in the P188-PhB shell and the acceptor in the PLGA nanoparticle core. Poloxamer 188 reacts with rhodamine B through the terminal hydroxyl groups of polyoxyethylene (Figure 1) [29].

**Figure 6:**
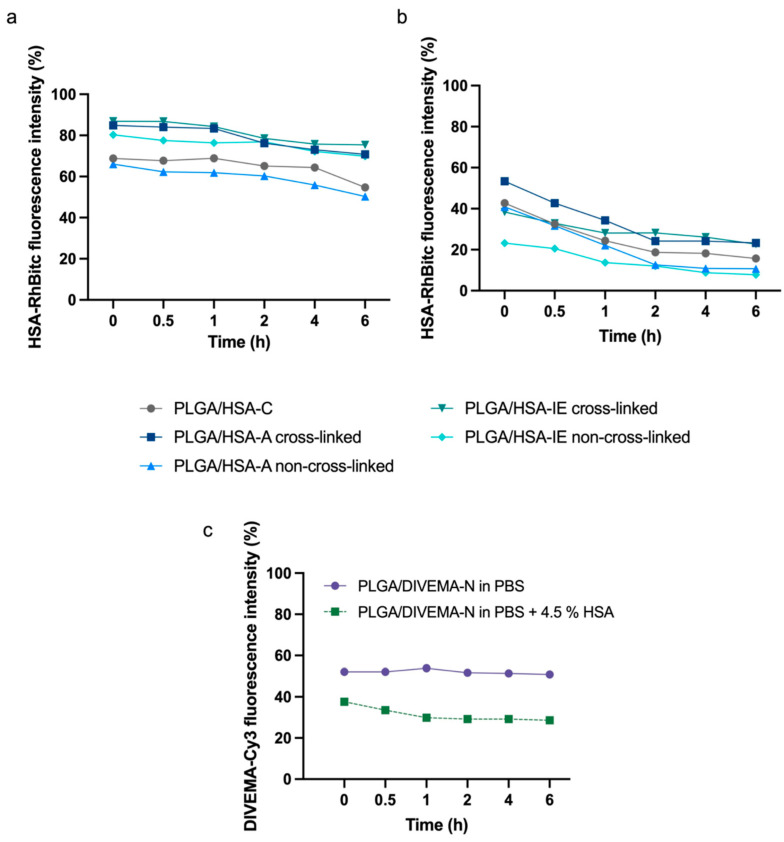
Evaluation of core–shell structure stability. Percentage of total shell-dye concentration remaining on the nanoparticles’ surface (based on relative fluorescence intensity values) after (**a**) 6 h of PLGA-Cy5/HSA-RhB incubation in PBS (pH 7.4); (**b**) 6 h of PLGA-Cy5/HSA-RhB incubation in PBS in the presence of 4.5% HSA (pH 7.4); and (**c**) 6 h of PLGA-Cy5/DIVEMA-Cy3 incubation in PBS in the presence or absence of 4.5% HSA (pH 7.4). Representative data.

To evaluate how the modification method influenced the core–shell structure’s stability, the amount of detached shell was assessed within 6 h of nanoparticle incubation in PBS (pH 7.4) and 4.5% HSA solution in PBS (pH 7.4) (imitating the HSA concentration in plasma). The amount of detached shell was evaluated based on the fluorescence intensity of the supernatant after the sample’s centrifugation at high acceleration for complete nanoparticle sedimentation (48,060× *g*). The stability of the core–shell structure was evaluated as the percentage of the shell remaining on the PLGA nanoparticles’ surfaces (Figure 6).

As shown in the graphs, considerable fractions of the HSA/RhBITC shells detached immediately upon their addition into the HSA-free medium for all nanoparticle types (Figure 6a). This initial burst effect was more pronounced for the PLGA/HSA NPs obtained through conjugation and adsorption without cross-linking (~30%). In contrast, in the case of the nanoparticles with cross-linked shells (adsorption-linked and interfacial-embedding-linked), more than 80% of the shell remained attached to the surface within 1 h of incubation (Figure 6a).

The HSA-RhBITC shells appeared to be less stable in the HSA-containing medium (4.5% HSA in PBS), with more than 50% of the shell detaching from the nanoparticle surface within the first hour (Figure 6b, Supplementary Table S4). Interestingly, the PLGA/HSA-IE non-cross-linked NPs, which were highly stable in albumin-free medium, demonstrated the lowest stability in the presence of 4.5% HSA in the medium. The initial burst effect (with a considerable amount of detached HSA-RhBITC) in all cases, including for the PLGA/HSA-C NPs obtained through conjugation, could be explained by the fast detachment of HSA, loosely associated with the surface. Predictably, the nanoparticles prepared with an additional cross-linking stage were more stable compared to the non-linked ones. Accordingly, the cross-linked nanoparticles (PLGA/HSA-IE cross-linked and PLGA/HSA-A cross-linked) were chosen for further in vitro experiments.

A similar trend (of more pronounced shell detachment in the presence of HSA) was observed for the PLGA/DIVEMA NPs (Figure 6c, Supplementary Table S4). In this case, the detachment of the loosely bound polyanion was facilitated by its interaction with the albumin abundantly present in the medium [55]. The amount of detached shell was evaluated based on the relative fluorescence intensity (DIVEMA-Cy3) of the supernatant after sample centrifugation.

The stability of the core–shell nanoparticles was also assessed at a more acidic pH, corresponding to the pH of late endosomes. No significant differences were found in shell detachment in more acidic (5.5) and neutral (7.4) pH environments in the presence of 4.5% HSA (Supplementary Table S5).

### 3.3. Investigation of the Integrity of the Core–Shell Nanoparticles upon Their Internalization into Gl261 Cells Using Confocal Microscopy

Prior to the cellular uptake experiments, the cytotoxicity of the core–shell PLGA/HSA, PLGA/DIVEMA, and PLGA/P188 nanoparticles was assessed in vitro in Gl261 cells using the MTS assay. Plain PLGA NPs without a shell were used as a reference. The reduction in cell viability after incubation with either the core–shell PLGA or plain PLGA NPs at concentrations of up to 100 μg/mL did not exceed 20%, which is consistent with previously obtained data [27]. As shown in [27], plain PLGA and PLGA/DIVEMA nanoparticles exhibited low cytotoxicity in LLC-PK1 porcine kidney epithelial cells and Hep G2 human hepatocellular carcinoma cells.

**Figure 7:**
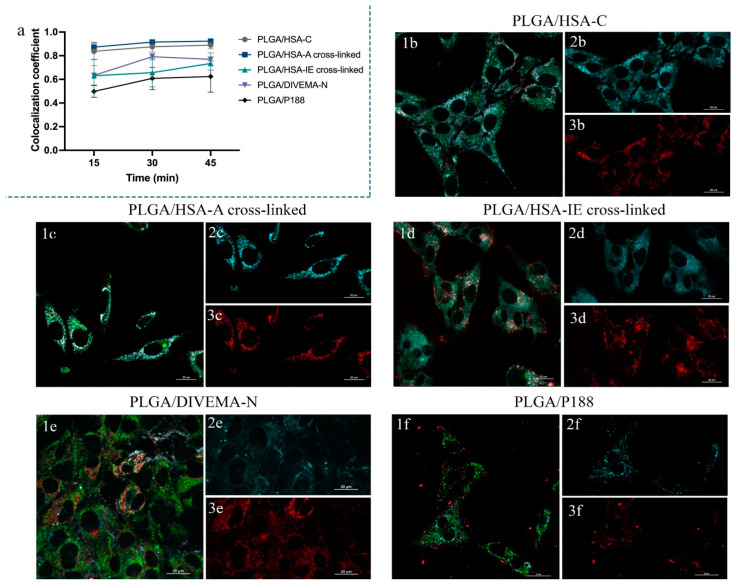
Confocal images of Gl261 cells after incubation with core–shell nanoparticles. (**a**) Manders’ overlap coefficients (MOCs) showing colocalization between the core and the shell of the nanoparticles at different time points (15, 30, and 45 min; n = 3); (**b**) PLGA/HSA-C; (**c**) PLGA/HSA-A cross-linked; (**d**) PLGA/HSA-IE cross-linked; (**e**) PLGA-DIVEMA-N; (**f**) PLGA/P188. For each type of nanoparticles (**b**–**f**) were shown: (1) Merged images (green—lysosomes; cyan—shell; red—core); (2) shell (HSA-RhBITC, DIVEMA-Cy3, or P188-RhB); and (3) core (PLGA-Cy5). CLSM. Scale bar: 20 μm.

To investigate how the modification method affected the integrity of the PLGA/HSA NPs (i.e., the colocalization of the core and the surface-bound albumin), the Manders’ overlap coefficients (MOCs) between the PLGA-Cy5 (core) and the HSA-RhBITC (shell) were calculated upon their incubation with the cells for different time periods (Figure 7a, Supplementary Table S5). Colocalization coefficients above 0.5 indicated a correlation between the nanoparticle core and shell localization in the course of the experiment for all nanoparticle types. However, the MOC values were significantly lower at all time points for the PLGA/HSA NPs obtained through interfacial embedding (0.63 compared to 0.83 and 0.87 after 15 min of incubation for the nanoparticles produced through HSA conjugation or adsorption, respectively; Figure 7a, Supplementary Table S6).

The intracellular internalization of the PLGA/HSA-C, PLGA/HSA-A cross-linked, and PLGA/HSA-IE cross-linked nanoparticles exhibiting higher stability in the model media was investigated in more detail (Figure 7, Supplementary Figures S9–S11). Interestingly, unlike the other PLGA/HSA nanoparticles (Figure 7b,c), the PLGA/HSA-IE cross-linked NPs tended to accumulate on the outer membrane after 15–30 min of incubation with the cells (Figure 7d), while the HSA-RhBITC signal was detected in the cell cytoplasm (Supplementary Figure S9). Subsequent incubation of this sample (30–45 min) led to increased colocalization between the core and the shell (0.73 after 45 min of incubation).

Similar experiments were performed for the nanoparticles coated with DIVEMA produced using the nanoprecipitation method (Figure 7e). The MOC values ranged from 0.63 to 0.79, suggesting that these nanoparticles preserved their core–shell structure (Supplementary Table S7). At 15 min, the MOC value was 0.63, indicating the presence of unbound or easily detached DIVEMA-Cy3. Indeed, a significant amount of free DIVEMA-Cy3 accumulated in the cell cytoplasm during the first few minutes of incubation (Supplementary Figure S12).

The P188 coating of the PLGA nanoparticles appeared to be less stable (Figure 7f, Supplementary Figure S13). During the first 15 min of incubation of the PLGA/P188 NPs with the cells, the MOC was 0.49 (Supplementary Table S8), indicating that the uptake of the free poloxamer by the cells occurred faster than the internalization of the PLGA nanoparticles. Further incubation (30–45 min) increased the average values of the colocalization coefficients, indicating a high level of overlap between the PLGA-Cy5 and P188-RhB signals. The colocalization coefficients varying from 0.49 to 0.62 during incubation with the cells suggested that the core–shell structure partially preserved its integrity upon nanoparticle internalization and intracellular trafficking.

The colocalization coefficients were also calculated between the PLGA-Cy5 nanoparticle cores and the lysosomes (Supplementary Tables S6–S8). Interestingly, the modification of the PLGA nanoparticles with different shells did not significantly alter the colocalization of the PLGA-Cy5 nanoparticle cores with the lysosomes in the Gl261 cells: the MOC values were found to vary from 0.3 to 0.5 for almost all samples, suggesting only partial accumulation of the nanoparticles in the lysosomes.

### 3.4. Investigation of the Dynamics of Nanoparticle Uptake by the Gl261 Glioma Cells

**Figure 8:**
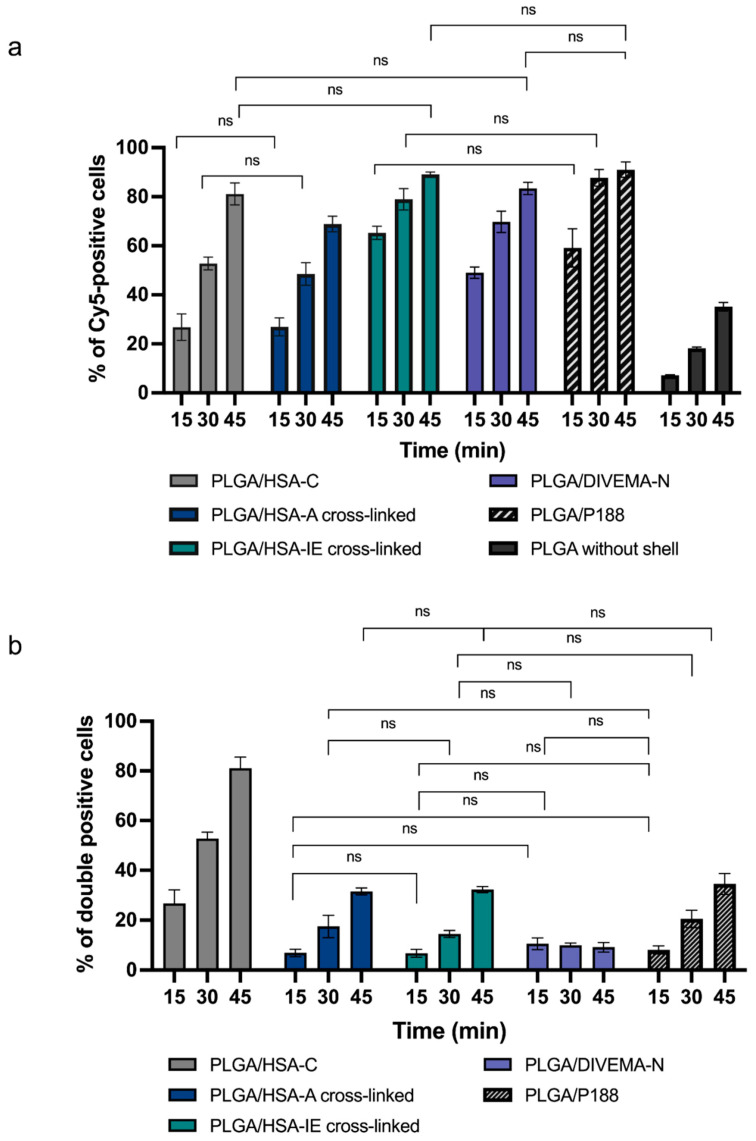
Core–shell nanoparticle uptake by Gl261 cells depending on the nanoparticle preparation technique and incubation time (15, 30, and 45 min of incubation). (**a**) % Cy5-positive cells and (**b**) % cells doubly positive for Cy5- and RhBITC/Cy3/RhB with PLGA-Cy5/HSA-RhBITC NPs, PLGA-Cy5/DIVEMA-Cy3 NPs, and PLGA-Cy5/P188-RhB NPs, respectively. Statistical analysis was performed using two-way ANOVA followed by Tukey’s multiple comparison test (mean ± SD, n = 3); ns = not significant; *p* > 0.05.

The nanoparticle uptake by the cells was determined as the percentage of NP-positive cells: PLGA-Cy5-positive or double-positive (both PLGA-Cy5- and HSA-RhBITC-, DIVEMA-Cy3-, or P188-RhB-positive) cells (Figure 8). First, the percentage of PLGA-Cy5 positive cells was calculated to evaluate the possible influence of surface modification of the nanoparticles on their interaction with cells. Then, the percentage of cells positive for both PLGA-Cy5 (the core) and HSA-RhBITC, RhB, or Cy3 (the shell) was determined to assess the structural integrity of the nanoparticles upon their interaction with the cells over time. The nanoparticle uptake by the cells (the percentage of nanoparticle-positive cells) tended to increase with time for the nanoparticles obtained using all shell-forming techniques.

The presence of the HSA shell increased the number of nanoparticle-positive cells (Figure 8a, Supplementary Table S9). The percentage of PLGA-Cy5-positive cells significantly increased compared to the control PLGA-Cy5 nanoparticles without shells (*p* < 0.0001 for all samples), regardless of the preparation method used (only statistically insignificant data are shown in the graph). This phenomenon could be attributed to the interaction of the nanoparticles with albumin-binding proteins on the cell membranes. Interestingly, based on the PLGA-Cy5 fluorescence (nanoparticle core), the maximal percentage of cellular uptake was observed for the PLGA-HSA NPs obtained using interfacial embedding: around 65% of the PLGA-Cy5-positive cells were detected after only 15 min of incubation, compared to 27% for the PLGA/HSA nanoparticles produced through conjugation or adsorption (*p* < 0.0001). However, the percentage of double-positive cells (positive for both PLGA-Cy5 and HSA-RhBITC) was the highest for the PLGA/HSA nanoparticles obtained through conjugation (Figure 8b). The number of double-positive cells was twice as high for the PLGA/HSA-C NPs compared to the other HSA-coated batches at all time points investigated. These results are consistent with those from the confocal experiments, which demonstrated the high stability of the core–shell structure for PLGA/HSA-C NPs.

Nanoparticles modified with P188 have been successfully used in several in vivo studies [30], suggesting that P188 surface modification is sufficient to significantly enhance nanoparticles’ interaction with cells. Indeed, coating the PLGA nanoparticles with poloxamer 188 increased the number of PLGA-Cy5-positive cells by about 45% compared to bare PLGA-Cy5 nanoparticles after 30 min of incubation with the cells (Figure 8a, Supplementary Table S10).

As shown in Figure 8a, the presence of the DIVEMA shell also significantly increased the number of PLGA-Cy5-positive cells (Figure 8a, Supplementary Table S11); however, the percentage of double-positive cells remained below 10% throughout the entire incubation period. These results are consistent with the stability tests performed in a 4.5% HSA solution, where a significant portion of the shell already detached from the nanoparticle surface within the first few minutes of incubation in the HSA-containing medium. It is worth mentioning that flow cytometry can measure the number of events but cannot distinguish whether the nanoparticle core or shell is internalized by the cells or merely accumulated on the cell membrane [56]. In contrast, confocal microscopy enables detailed investigation of nanoparticle uptake, intracellular localization, and trafficking. Therefore, it is essential to use several methods, such as flow cytometry in conjunction with confocal microscopy, to fully characterize the behavior of nanoparticle formulations.

## 4. Discussion

The main objective of this study was to develop an integrated approach to the evaluation of core–shell nanoparticle stability using PLGA nanoparticles with different polymeric shells as the model structures. The objective of this study was to compare the properties of core–shell nanoparticles with a PLGA core and shells made from different types of polymers, focusing on their structural integrity. The core PLGA nanoparticles were prepared using either the high-pressure homogenization–solvent evaporation technique or nanoprecipitation. As mentioned above, the following shell-forming polymers were used: (1) poloxamer 188 (P188), a non-ionic triblock copolymer of polyethylene oxide and polypropylene oxide; (2) human serum albumin (HSA), one of the most abundant plasma proteins with a high ligand-binding capacity and a natural carrier of many exogenous and endogenous substances [57]; and (3) a copolymer of divinyl ether and maleic anhydride (DIVEMA), a polyanion with versatile biologic activity [58]. DIVEMA is a polyanhydride that, upon hydrolysis, turns into a polycarboxylate containing four carboxyl groups per monomeric unit. The shells were formed through adsorption, interfacial embedding, or conjugation.

For dual fluorescent labeling, the core- and shell-forming polymers were conjugated with Cyanine5, Cyanine3, and rhodamine B. The nanoparticles exhibited negative zeta potentials and sizes ranging from 100 to 250 nm (as measured using dynamic light scattering), depending on the shell structure and preparation method. The core–shell structure was confirmed through transmission electron microscopy (TEM) and fluorescence spectroscopy, with the appearance of Förster resonance energy transfer (FRET) phenomena due to the donor–acceptor properties of the fluorescent labels.

All of the shells enhanced the cellular uptake of the nanoparticles in Gl261 murine glioma cells. The integrity of the core–shell structure upon incubation with the cells was demonstrated by the intracellular colocalization of the fluorescent labels, measured using the Manders’ colocalization coefficients. This comprehensive approach provides valuable insight for selecting the optimal preparation method at the early stages of core–shell nanoparticle development.

The methods of shell formation were chosen based on the polymer properties. In the case of HSA, three techniques were used for shell formation to reveal the optimal conditions for the use of this versatile and complex molecule. HSA is known to adsorb onto various surfaces depending on their charge and hydrophobicity [59]; it can also provide steric stabilization of the colloidal carriers, protecting them from recognition by macrophages [60]. In the case of the adsorption technique, shell formation was achieved by incubating the “bare nanoparticles” in the HSA solution. For the interfacial embedding of HSA, the PLGA NPs were also prepared using the high-pressure homogenization–solvent evaporation technique; however, in this case, HSA (0.5% solution) was used as the stabilizer in the outer aqueous phase instead of PVA. Indeed, due to its amphiphilic properties, HSA can be located in the interfacial area between the organic and aqueous phases, with its hydrophilic fragments protruding into the water, while the more hydrophobic fragments are “immersed” into the PLGA core. As shown in Table 1, the method of interfacial embedding was the most effective, producing a considerably more abundant shell on the nanoparticle surfaces compared to HSA adsorption or conjugation (0.52, 0.11, and 0.48 mg HSA per mg of PLGA, respectively). Similar results were obtained in the study by Hyun at el., where the interfacial embedding of HSA yielded nanoparticles with a higher amount of HSA compared to HSA adsorption [49]. For better stability, the HSA shells in the PLGA/HSA NPs prepared through adsorption and interfacial embedding were additionally cross-linked using glutaric aldehyde. This approach was shown to improve the stability of the HSA-coated nanoparticles upon dilution and in the presence of other proteins (endogenous albumin) [61].

Interfacial embedding was also applicable to the preparation of PLGA/DIVEMA NPs. Indeed, the radical copolymerization of maleic anhydride and divinyl ether yields DIVEMA in its polyanhydride form (Figure 1). In this form, DIVEMA is readily soluble in acetone but not in water. When added into the aqueous–organic mixture during nanoparticle formation, the anhydride cycles are gradually hydrolyzed, converting the copolymer into an amphiphilic molecule. This process is rather slow, so the DIVEMA-Cy3 conjugate retains its amphiphilic nature and is capable of interacting with PLGA on the one hand and creating a hydrophilic shell on the nanoparticle surface on the other [62]. The presence of DIVEMA in the PLGA/DIVEMA nanoparticles was previously confirmed using FTIR spectroscopy [27]. Moreover, the presence of the outer shell rich in carboxyl groups on the PLGA/DIVEMA NPs’ surfaces was confirmed by the pH-dependent changes in their size, which decreased upon lowering the pH level or in the presence of salts due to the decreased dissociation of DIVEMA’s carboxyl groups. Both phenomena are typical of polyelectrolytes [27,51,63].

The presence of the shells was also confirmed through comparison of the nanoparticle sizes measured using TEM and DLS. Thus, the majority of the core–shell nanoparticles (with the exception of the PLGA/P188 NPs) had greater hydrodynamic diameters (Table 1, Figure 4) than the diameters measured using TEM (Figure 3). Accordingly, this phenomenon is most likely explained by the hydration of the hydrophilic shell in the aqueous medium.

The stability of a shell–core structure in a biological environment is an important factor influencing the performance of such delivery systems. As shown by the DLS measurements, all of the PLGA/HSA NPs and PLGA/DIVEMA-N NPs maintained their initial hydrodynamic diameters and polydispersity for at least 1 h in the biorelevant medium containing HSA (pH 7.4, PBS buffer with 4.5% of HSA), whereas in the albumin-free medium (PBS, pH 7.4), they were stable for at least 6 h (Figure 4, Supplementary Tables S1 and S2). An increase in the average particle size (by volume) occurred in the presence of HSA due to the adsorption of an excessive amount of HSA (PLGA/HSA NPs) or the replacement of the shell with HSA molecules (PLGA/DIVEMA NPs) from the medium, followed by particle aggregation, which was also observed by other authors [64]. The formation of a protein corona on PLGA/HSA NPs (adsorption and interfacial embedding) during nanoparticle incubation in serum was confirmed in [49].

Another parameter of the core–shell structure’s stability is the amount of the shell-forming polymer that remains associated with the core upon 6 h of incubation in the HSA-containing medium. The results of this test confirmed the lower stability of the PLGA/HSA systems in the presence of has, where the percent of the shell remaining on the nanoparticles’ surfaces ranged from ~20% to ~40% and ~8% to ~20% after 1 h and 6 h, respectively, whereas in the HSA-free medium, the respective values reached 60–87% and 50–75% (Figure 6a,b, Supplementary Table S4). As expected, the nanoparticles with cross-linked shells were more stable compared to the non-linked ones. A lower stability in the presence of HSA was also observed for the PLGA/DIVEMA NPs (Figure 6c, Supplementary Table S4). Apparently, the high initial amount of detached DIVEMA-Cy3 resulted from the shell being loosely associated with the surface. When entering the intercellular space of the tumor and being internalized into cells, the core–shell nanoparticles encounter an environment with a lower pH compared to that of plasma. While the pH of plasma and the cytoplasm is neutral (~7.4), the pH of the endosomal and lysosomal compartments varies: early endosomes have a pH of around 6.5, late endosomes have a pH of about 5.5, and lysosomes are highly acidic, with a pH of approximately 4.5 [65]. It was found that lowering the pH of the model medium from 7.4 to 5.5 with 4.5% HSA did not lead to an increase in the rate of membrane detachment, indicating that protein interactions, rather than acidity alone, play a more significant role in destabilizing the structure (Supplementary Table S5).

The core–shell integrity was unequivocally confirmed using fluorescence spectroscopy. Labeling of the cores and the shells of the nanoparticles with pairs of fluorescent dyes exhibiting donor–acceptor properties (Supplementary Figure S7) and therefore capable of the distance-dependent Förster resonance energy transfer (FRET) phenomenon enabled an evaluation of the closeness of the cores and shells. Indeed, as mentioned above, donor–acceptor pairs are capable of exhibiting the distance-dependent Förster resonance energy transfer (FRET) phenomenon, observed only in the case of close proximity (<10 nm) of both dye molecules, enabling the energy transfer from the donor fluorophore to the acceptor fluorophore [53,66,67]. The FRET phenomenon was exhibited by all types of PLGA/HSA nanoparticles, indicating the closeness of the shell and the PLGA core (interaction between RhBITC and Cy5), with the most prominent effect observed for the HSA shells obtained using the interphase embedding method (Supplementary Figure S8). On the other hand, the absence of the FRET phenomenon in the case of the PLGA/P188 NPs could be due to the orientation of this amphiphilic block copolymer. Indeed, when P188-RhB is adsorbed onto the surface of PLGA nanoparticles, the hydrophobic segments of polyoxypropylene most likely interact with the hydrophobic core of PLGA, while the hydrophilic chains of polyoxyethylene with the grafted dye are solubilized by water molecules and protrude into the surrounding medium [68,69], which prevents them from approaching the nanoparticle surface and hinders efficient energy transfer from the donor to the acceptor.

One of the main objectives of this study was to track the integrity of the core–shell structures during their incubation with the cell culture, thus providing a more comprehensive overview of the nanoparticles’ structural changes (lifecycle) upon their interaction with the cells. Dual labeling of the nanoparticles allowed for evaluation of the core–shell integrity over time using confocal laser scanning microscopy (CLSM). Live-cell imaging was performed at different time intervals during the nanoparticles’ incubation with the Gl261 cells. The Manders’ overlap coefficient (MOC) was chosen for evaluating the core–shell colocalization, as it is widely used in fluorescence microscopy to quantify colocalization and is implemented in most biological image analysis software packages [70]. Unlike Pearson’s correlation, the MOC is primarily sensitive to the co-occurrence of signals from two channels, almost independent of the signal level and proportionality. The colocalization between the core and the shell was observed for all the types of nanoparticles (MOC > 0.50, Figure 7a, Supplementary Tables S6–S8). The lower MOC value for the PLGA/HSA-IE cross-linked NPs, compared to the other HSA shells, may have been due to the partial detachment of the shells from the cores upon their introduction into the cell culture medium due to dilution, suggesting that these nanoparticles are less stable upon internalization into cells than those obtained through conjugation or adsorption (Supplementary Figures S10 and S11).

It is noteworthy that the presence of the shells did not particularly influence the trafficking of the PLGA NPs into the lysosomes, which was ≤50% for all nanoparticle types (MOC values between the PLGA-Cy5 nanoparticle core and lysosomes of 0.3–0.5; Supplementary Tables S6–S8). Late endosomes and lysosomes are the main compartments involved in clathrin-mediated endocytosis, which is known to be one of the most common pathways for polymeric nanoparticle internalization in the vast majority of cell cultures [71]. Thus, it can be hypothesized that clathrin-mediated endocytosis is not the main internalization route for PLGA nanoparticles. These data align with the results of Beigulenko et al., who demonstrated that only a small portion of PLGA nanoparticles coated with fluorescently labeled poloxamer (P188-RhB) associated with lysosomes [29]. It is also worth mentioning that caveolin-mediated endocytosis plays the primary role in albumin uptake into cells [72]. Therefore, albumin coating on nanoparticles could be expected to affect the intracellular trafficking of PLGA nanoparticles. However, this was not observed in the present study. One possible reason for this could be a change in the conformation of the albumin molecules on the nanoparticles’ surfaces, leading to alterations in the interaction between the nanoparticles and cells [49]. These findings, however, require further investigation of the endocytosis routes, as they may also be attributed to the specific characteristics of the cell culture.

To gain a deeper understanding of the nanoparticles’ interactions with the cells, the dynamics of their accumulation in the Gl261 cells was investigated using flow cytometry. Thus, despite the different nature and properties of the shell-forming polymers, all of the shells enhanced the nanoparticle uptake by the Gl261 cells as compared to that of the uncoated nanoparticles, exhibited as increased numbers of nanoparticle-positive cells (Figure 8a, Supplementary Tables S9–S11). In the case of the PLGA/HSA nanoparticles, the highest stability of the HSA shells upon incubation in the HSA-containing media and with the Gl261 cells was achieved through conjugation (PLGA/HSA-C), compared to the adsorption (PLGA/HSA-A) and interfacial embedding methods (PLGA/HSA-IE). Interestingly, the interfacial embedding method provided the highest shell content (HSA, DIVEMA) per weight of polymer (~0.5 mg/mg PLGA) and a high FRET effect; however, in both cases, the low percentage of core–shell-double-positive cells (in flow cytometry) and the low Manders’ overlap coefficient (in CLSM) indicated a loss of core–shell integrity in the presence of HSA in the incubation medium. The differences between the core–shell systems may become more pronounced in in vivo conditions when they are introduced into a very dynamic multicomponent environment and various blood components compete for the space on the particles’ surfaces. At the same time, the suggested combination of evaluation techniques enabled a thorough analysis of the core–shell structures’ stability and may be useful for the selection of the optimal preparation method even at the early stages of nanoparticle development.

This ability of the core–shell structures to enhance the nanoparticle uptake into the cells and for their shells to be, at least partially, preserved upon their internalization suggests that the shells may also be functional in in vivo conditions. This hypothesis is supported by the results of other authors. In the study by Hyun et al., the HSA shells being cross-linked using dopamine facilitated the uptake of the PLGA nanoparticles by tumors [49]. In their classical studies, L. Illum and S.S. Davis demonstrated that despite the in vitro displacement of adsorbed poloxamine and poloxamer by serum proteins on the surface of PLGA nanoparticles [73], these coatings still exerted a profound influence on the in vivo biodistribution of the nanoparticles [74].

Thus, although the scope of this study was limited to an in vitro evaluation of the stability of core–shell nanoparticles, the suggested approach may help to identify the most stable nanoparticle candidates for further in vivo studies, thereby minimizing the risk of potential artifacts. Through this evaluation, we aimed to identify the most promising and reliable formulations, paving the way for more accurate and reproducible results in future in vivo studies.

## 5. Conclusions

In this study, the properties of core–shell nanoparticles with a PLGA core and shells made of polymers with significantly different structures and physicochemical properties were compared, with a focus on their structural integrity. The basic parameters of the nanoparticles, such as their size and zeta potential, depended on the shell composition and preparation technique; however, all of the particles had hydrodynamic diameters of ≤250 nm and were negatively charged—parameters considered optimal for drug delivery. Notably, despite differences in their chemical structures, polarity, molecular mass, and hydrophobicity, all of the shells were largely retained by the core nanoparticles in both biorelevant media and within cells. Moreover, all of the shells enhanced the cellular uptake of the nanoparticles. The structural integrity of the core–shell PLGA nanoparticles was confirmed using a variety of methods, including physicochemical evaluation, fluorescence spectroscopy, confocal microscopy, and flow cytometry. The latter methods were facilitated by dual labeling of the nanoparticles with dyes possessing donor–acceptor properties. This comprehensive approach provides valuable insights into the behavior of core–shell nanoparticles in different environments and their interactions with tumor cells.

## Figures and Tables

**Table 1:** Physicochemical parameters of the nanoparticles (mean ± SD, n = 3).

Method of Shell Formation (Method of Nanoparticle Preparation *)	Nanoparticle Size and Size Distribution, nm			Zeta Potential, mV	Shell Content, mg/mg PLGA
	Mean Diameter	PDI	Volume Size Distribution		
PLGA-Cy5/HSA-RhBITC NP					
Conjugation (PLGA/HSA-C)	153 ± 2	0.201 ± 0.017	147 (100%)	−7.0 ± 1.2	0.48
Adsorption, cross-linked (PLGA/HSA-A cross-linked)	148 ± 2	0.183 ± 0.018	143 (100%)	−7.6 ± 0.8	0.11
Adsorption, non-cross-linked (PLGA/HSA-A non-cross-linked)	135 ± 1	0.118 ± 0.014	133 (100%)	−6.2 ± 2.7	0.11
Interfacial embedding, cross-linked (PLGA/HSA-IE cross-linked)	103 ± 3	0.138 ± 0.014	92 (100%)	−26.3 ± 0.7	0.50
Interfacial embedding, non-cross-linked (PLGA/HSA-IE non-cross-linked)	90 ± 1	0.056 ± 0.022	83 (100%)	−31.9 ± 2.7	0.52
PLGA without a shell ***	116 ± 2	0.098 ± 0.015	108 (100%)	−20.9 ± 1.1	-
PLGA-Cy5/DIVEMA-Cy3 NP					
Interfacial embedding (PLGA/DIVEMA-H)	264 ± 4	0.225 ± 0.009	341 (94.2%) 5118 (5.8%)	−34.9 ± 0.1	0.10
PLGA without shell **	178 ± 24	0.230 ± 0.050	290 (100%)	−20.3 ± 1.7	-
Interfacial embedding (nanoprecipitation) (PLGA/DIVEMA-N)	180 ± 21	0.274 ± 0.007	284 (100%)	−51.3 ± 1.4	0.47
NPs without a shell (nanoprecipitation) **	130 ± 15	0.070 ± 0.015	170 (100%)	−18.5 ± 3.5	-
PLGA-Cy5/P188-RhB					
Adsorption of P188-RhB	97 ± 1	0.100 ± 0.007	85 (100%)	−21.8 ± 0.7	0.017
Adsorption of P188	110 ± 2	0.16 ± 0.01	97 (100%)	−23.5 ± 1.1	0.006
PLGA without a shell ***	100 ± 2	0.078 ± 0.015	100 (100%)	−20.9 ± 1.1	-

* The nanoparticles were prepared through the high-pressure homogenization–solvent evaporation technique unless indicated otherwise. ** The nanoparticles were prepared with PVA as a surfactant. *** Plain NPs were used to obtain core–shell particles through adsorption or conjugation methods.

**Table 2:** Fluorescent properties of core–shell nanoparticles: representative data.

Sample	Dye Content, µg/mL		Dye-to-Polymer Ratio, µg/mg		Quantum Yield		Brightness, M^−1^ cm^−1^	
PLGA-Cy5/ HSA-RhBITC	RhBITC	Cy5	RhBITC	Cy5	RhBITC	Cy5	RhBITC	Cy5
PLGA/HSA-C	4.80	1.62	3.93	0.64	0.09	0.12	1.03 × 10^8^	3.61 × 10^7^
PLGA/HSA-A cross-linked	3.62	1.81	12.07	0.68	0.11	0.18	3.78 × 10^7^	6.70 × 10^7^
PLGA/HSA-A non-cross-linked	4.86	2.53	16.20	0.88	0.15	0.23	3.41 × 10^7^	6.46 × 10^7^
PLGA/HSA-IE cross-linked	3.11	1.31	0.98	0.78	0.19	0.31	1.42 × 10^8^	1.46 × 10^7^
PLGA/HSA-IE non-cross-linked	2.64	1.80	0.80	0.85	0.21	0.29	9.53 × 10^7^	1.01 × 10^7^
PLGA-Cy5/ DIVEMA-Cy3	Cy3	Cy5	Cy3	Cy5	Cy3	Cy5	Cy3	Cy5
	4.12	2.36	11.4	2.03	0.12	0.21	1.66 × 10^8^	2.56 × 10^8^
PLGA-Cy5/P188-RhB	RhB	Cy5	RhB	Cy5	RhB	Cy5	RhB	Cy5
	4.26	4.65	44.8	0.83	0.63	0.37	3.77 × 10^7^	4.19 × 10^7^

## Data Availability

The data will be made available on request.

## Supplementary Materials

The following supporting information can be downloaded at https://www.mdpi.com/article/10.3390/biom14121601/s1. Figure S1: FTIR spectra: DIVEMA-Cy3 conjugate; Figure S2: FTIR spectra: PLGA-Cy5 conjugate; Figure S3: FTIR spectra: HSA-RhBITC; Figure S4: FTIR spectra: P188-RhB; Figure S5: Schematic representation of the preparation of dual-labeled nanoparticles with a PLGA-Cy5 core and various shells; Figure S6: Images of aqueous suspensions of core–shell nanoparticles; Figure S7: Normalized spectra of potential FRET pairs using Cy5 as the acceptor; Figure S8: Fluorescent spectra of PLGA-Cy5/HSA-RhBITC NPs in water; Figure S9: Confocal images of Gl261 cells after incubation with PLGA/HSA-IE cross-linked NPs; Figure S10: Confocal images of Gl261 cells after incubation with PLGA/HSA-A-C NPs; Figure S11: Confocal images of Gl261 cells after incubation with PLGA/HSA-A cross-linked NPs; Figure S12: Confocal images of Gl261 cells after incubation with PLGA/DIVEMA-N NPs; Figure S13: Confocal images of Gl261 cells after incubation with PLGA/P188 NPs; Table S1: The size and size distribution of core–shell nanoparticles upon 6 h of incubation in PBS in the presence of 4.5% HSA; Table S2: The size and size distribution of core–shell nanoparticles upon incubation in PBS; Table S3: Fluorescent properties of free dyes; Table S4: Estimation of the percentage of shell remaining on the nanoparticle surface after 6 h of incubation in PBS in the presence and absence of 4.5% HSA, pH = 7.4; Table S5: Estimation of the percentage of shell remaining on the nanoparticle surface after 6 h of incubation in PBS in the presence of 4.5% HSA, pH = 5.5; Table S6: Colocalization between the PLGA-Cy5 core and an HSA-RhBITC shell and lysosomes in Gl261 cells; Table S7: Colocalization between the PLGA-Cy5 core and a DIVEMA-Cy3 shell and lysosomes in Gl261 cells; Table S8: Colocalization between the PLGA-Cy5 core and a P188-RhB shell and lysosomes in Gl261 cells; Table S9: Evaluation of PLGA-Cy5/HSA-RhBITC NP uptake by Gl261 cells at different time points; Table S10: Evaluation of PLGA-Cy5/P188-RhB uptake by Gl261 cells at different time points; Table S11: Evaluation of PLGA-Cy5/DIVEMA-Cy3 NP uptake by Gl261 cells at different time point.
