# Supplementary material for: Core–Shell PLGA Nanoparticles: In Vitro Evaluation of System Integrity
Source: Biomolecules. 2024 Dec 14;14(12):1601. doi: 10.3390/biom14121601 (PMC11674307; doi:10.3390/biom14121601)
Supplement: Supplementary file 1 [file biomolecules-14-01601-s001.zip › biomolecules-3310670-supplementary.pdf]

Supplementary Materials to the Article:

Core-shell PLGA nanoparticles: *in vitro* evaluation of system integrity

Tatyana Kovshova, Julia Malinovskaya, Julia Kotova, Marina Gorshkova, Lyudmila Vanchugova, Nadezhda Osipova, Pavel Melnikov, Veronika Vadekhina, Alexey Nikitin, Yulia Ermolenko, Svetlana Gelperina

FTIR spectroscopy study

Fourier transform infrared (FTIR) spectroscopy of the polymer-dye conjugates and the reference samples were registered using a PerkinElmer Spectrum One FTIR spectrometer equipped with universal ATR Sampling Accessories with Diamond/ZnSe crystals (PerkinElmer Inc., USA).

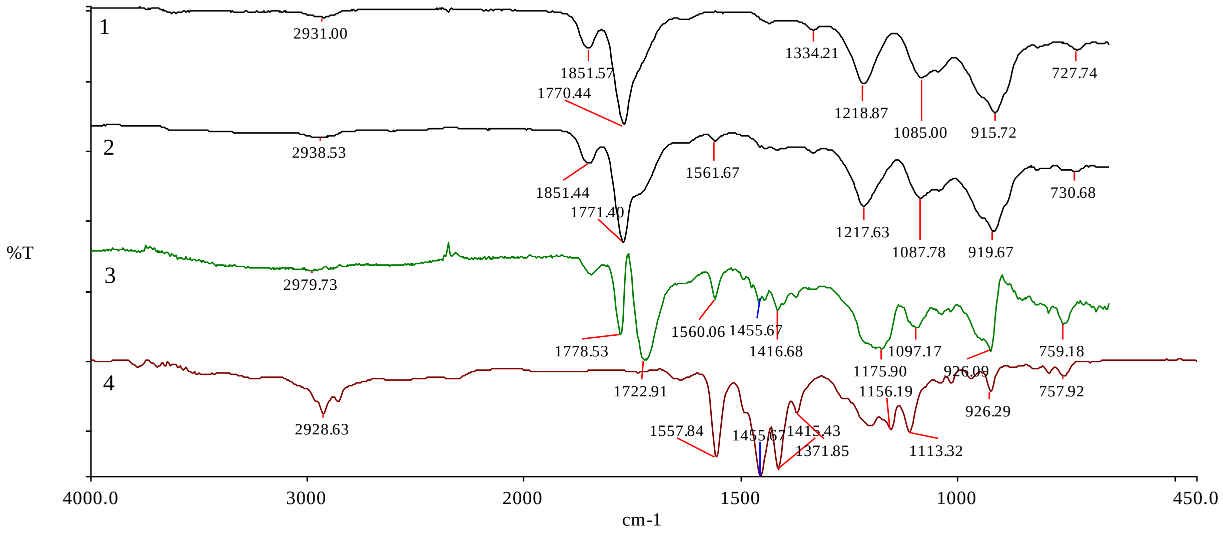

Figure S1. FTIR spectra: DIVEMA-Cy3 conjugate. 1 - DIVEMA-Cy3 conjugate, 2 - differential spectrum of the DIVEMA-Cy3 conjugate, 4 - Cyanine3.

The formation of an amide bond as a result of condensation of the dye amino groups with the acid groups of DIVEMA was evidenced by the presence in the spectrum of the conjugate of a band - amide II in the region of 1560 cm^{-1}, related to the structural fragment C-N-H. This band was clearly visible in the differential spectrum (3), which also contained bands related to the dye: 2928 cm^{-1} - CH vibrations of aliphatic groups; stretching vibrations of the C-N bond at 1450 cm^{-1} and N-H bonds cm^{-1} in the region of 760 cm^{-1}. Thus, the IR Fourier spectroscopy data confirmed the presence of a dye attached by a covalent amide bond in the DIVEMA-Cy3 conjugate.

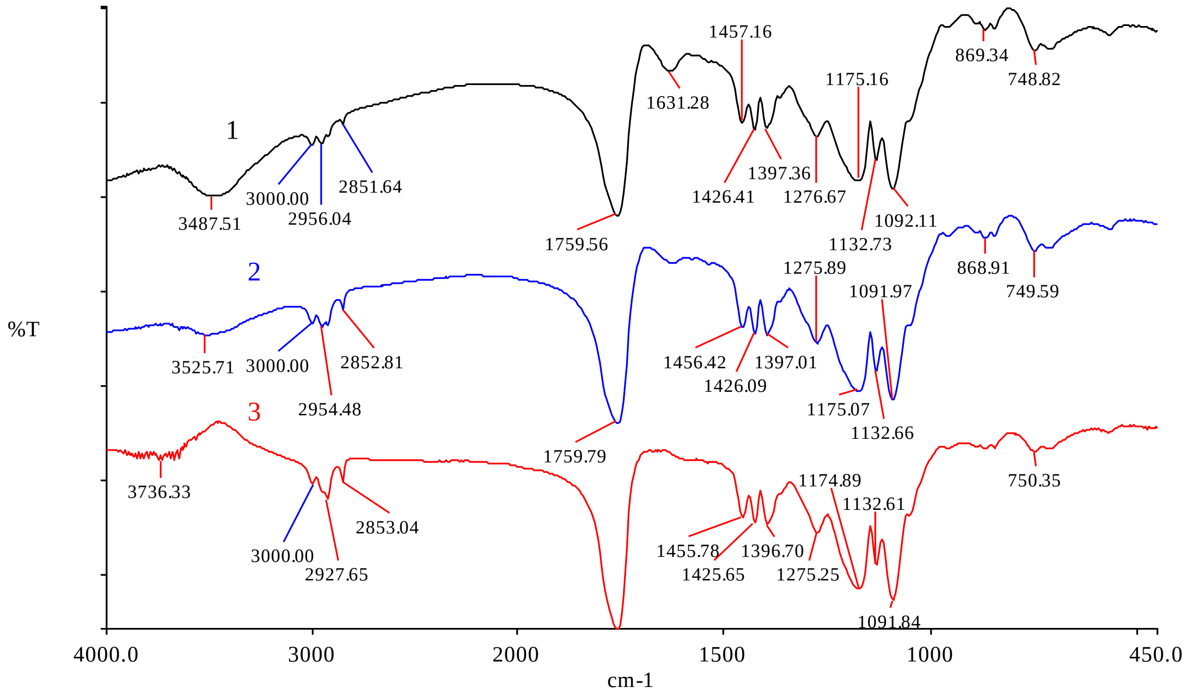

Figure S2. FTIR spectra: PLGA-Cy5 conjugate. 1 - PLGA, 2 - PLGA-Cy5 conjugate, 3 - differential spectrum PLGA-Cy5 - PLGA.

Increased intensity of the bands at 2800-2900 cm^{-1} may be due to the presence of aliphatic groups of Cy5.

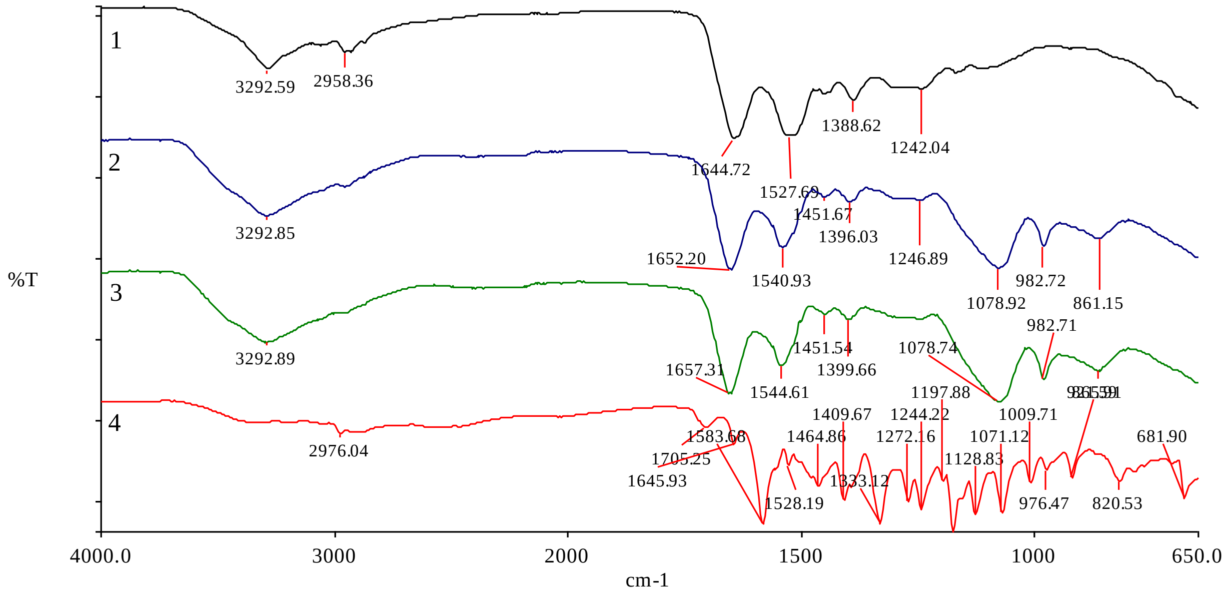

Figure S3. FTIR spectra: HSA-RhBITC conjugate. 1 – HSA, 2 - HSA-RhBITC conjugate, 3 – differential spectrum HSA-RhBITC - HSA; 4 - RhBITC.

In the differential spectrum, a shift of the amide II C-N band is observed in the region of 1520-1540 cm-1. However, against the background of the albumin spectrum bands, it is difficult to make an unambiguous conclusion about the presence of a new albumin-dye amide bond.

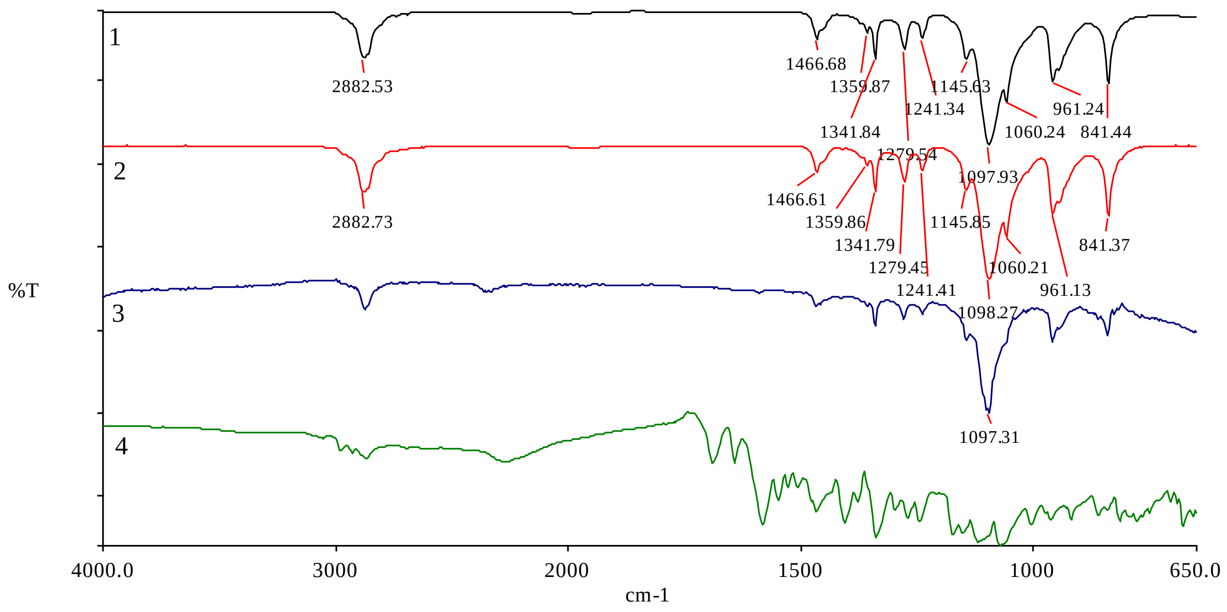

Figure S4. FTIR spectra: P188-RhB conjugate. 1 - P188; 2 - P188-RhB conjugate, 3 - RhB; 4 - differential spectrum P188-RhB conjugate - P188.

In the differential spectrum, the C=C bond of aromatic rings is observed at 1590 cm^{-1}. However, the ester band that is typically present at 1720 cm^{-1} is not visible.

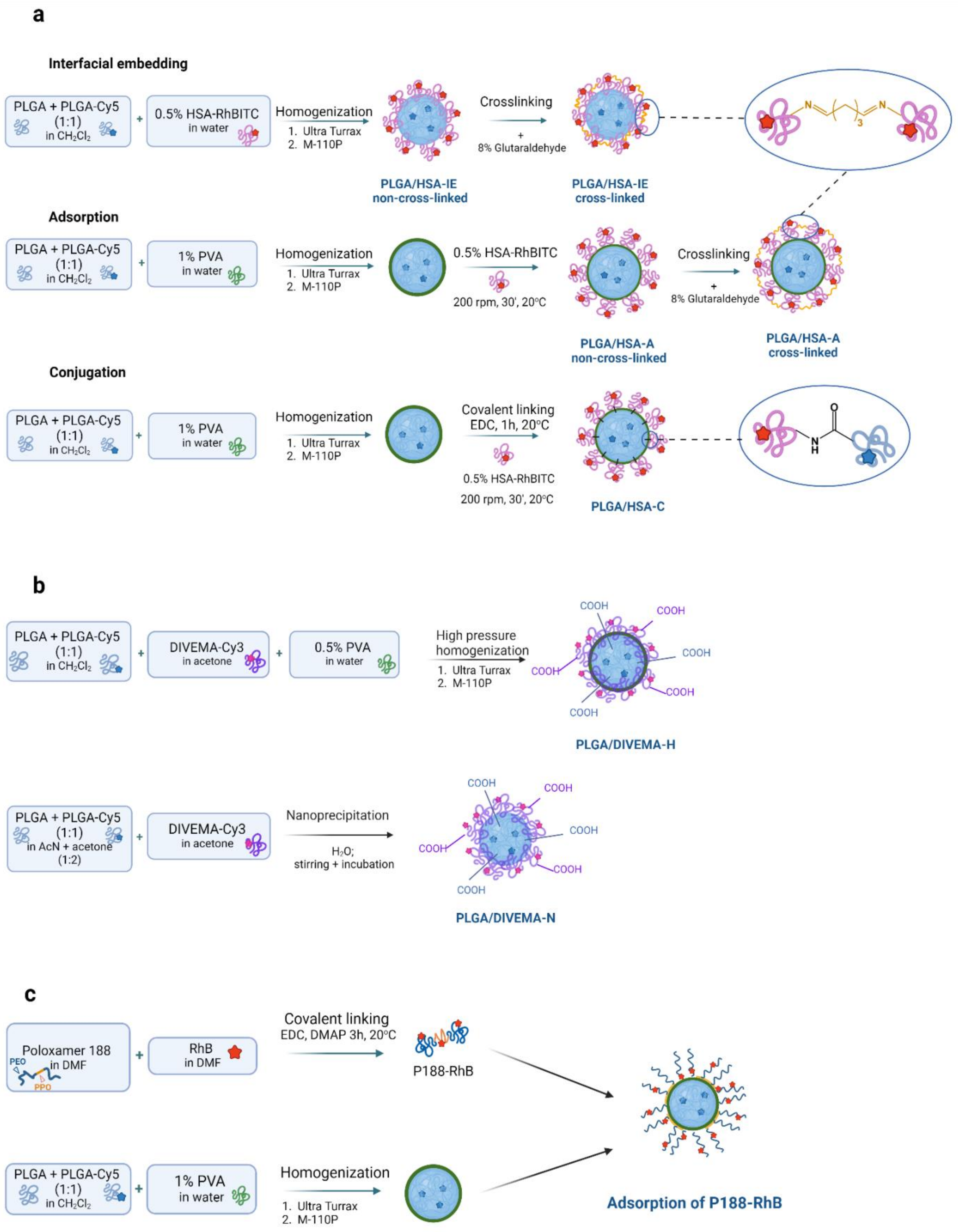

Figure S5. Schematic representation of the preparation of dual labeled nanoparticles with a PLGA-Cy5 core and various shells. (a) RhBITC-labeled HSA shell obtained by different techniques; (b) Cy3-labeled DIVEMA shell; (c) RhB-labeled P188 shell. Created with BioRender.com.

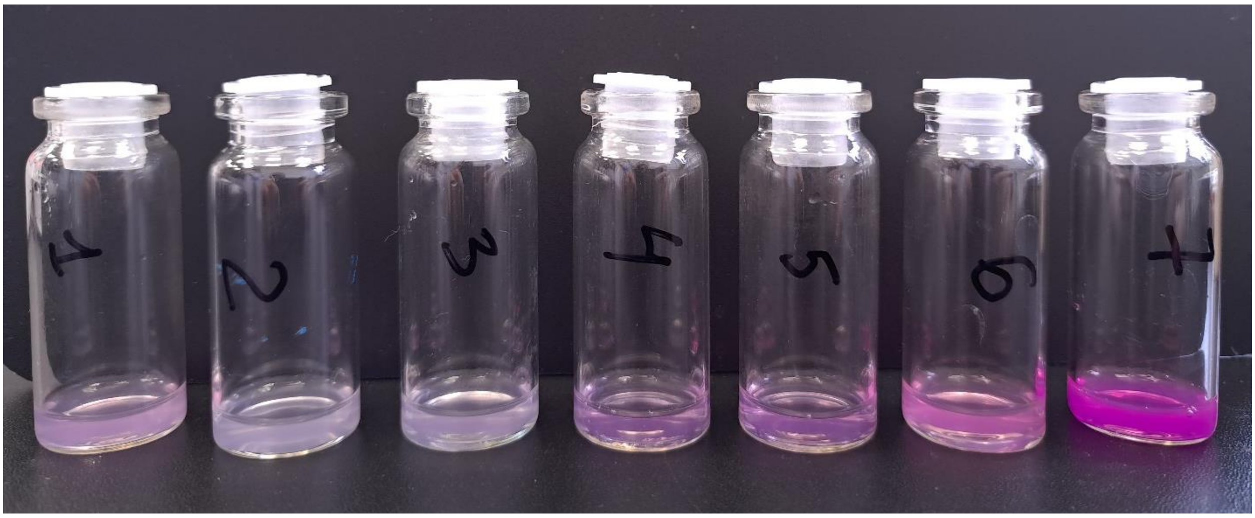

Figure S6. Images of aqueous suspensions of core-shell nanoparticles. (1) PLGA/HSA-C; (2) PLGA/HSA-A cross-linked; (3) PLGA/HSA-A non-cross-linked; (4) PLGA/HSA-IE cross-linked; (5) PLGA/HSA-IE non-cross-linked; (6) PLGA/DIVEMA-N; (7) PLGA-Cy5/P188-RhB.

Table S1. The size and size distribution of core-shell nanoparticles upon 6 hours of incubation in PBS in the presence of 4.5% HSA. Mean ± SD, n=3.

Nanoparticle type	Time, h	Nanoparticle size and size distribution		
		Mean diameter. nm	PDI	Volume size distribution, nm
**PLGA/HSA-C**	0	109 ± 2	0.395 ± 0.050	91 ± 3 (95%)
	0.5	100 ± 1	0.380 ± 0.060	92 ± 2 (94%)
	1	99 ± 4	0.490 ± 0.036	90 ± 6 (92%)
	2	129 ± 1	0.386 ± 0.007	135 ± 2 (100%)
	4	159 ± 1	0.386 ± 0.007	150 ± 2 (97%)
	6	205 ± 4	0.478 ± 0.079	280 ± 7 (100%)
**PLGA/HSA-A cross-linked**	0	99 ± 2	0.400 ± 0.002	90 ± 5 (97%)
	0.5	98 ± 2	0.380 ± 0.003	92 ± 4 (95%)
	1	100 ± 1	0.420 ± 0.090	93 ± 2 (91%)
	2	121 ± 1	0.297 ± 0.010	125 ± 3 (97%)
	4	156 ± 1	0.244 ± 0.023	158 ± 3 (97%)
	6	221 ± 2	0.571 ± 0.032	148 ± 3 (95%)
**PLGA/HSA-A non-cross-linked**	0	95 ± 1	0.300 ± 0.005	91 ± 4 (100%)
	0.5	99 ± 1	0.297 ± 0.006	90 ± 2 (96%)
	1	90 ± 1	0.300 ± 0.009	85 ± 3 (95%)
	2	100 ± 2	0.350 ± 0.050	99 ± 3 (100%)
	4	238 ± 6	0.371 ± 0.019	235 ± 9 (99%)
	6	298 ± 3	0.380 ± 0.010	305 ± 6 (98%)
**PLGA/HSA-IE cross-linked**	0	103 ± 3	0.280 ± 0.050	125 ± 7 (100%)
	0.5	105 ± 3	0.295 ± 0.105	109 ± 7 (100%)
	1	109 ± 4	0.360 ± 0.040	110 ± 9 (100%)
	2	118 ± 2	0.394 ± 0.020	120 ± 5 (94%)
	4	118 ± 4	0.538 ± 0.044	99 ± 6 (91%)
	6	123 ± 4	0.500 ± 0.058	146 ± 7 (90%)
**PLGA/HSA-IE non–cross-linked**	0	97 ± 3	0.395 ± 0.100	99 ± 6 (100%)
	0.5	99 ± 3	0.400 ± 0.050	98 ± 5 (100%)
	1	95 ± 4	0.380 ± 0.030	99 ± 8 (100%)
	2	94 ± 1	0.387 ± 0.021	84 ± 4 (96%)
	4	99 ± 1	0.474 ± 0.098	89 ± 2 (95%)
	6	125 ± 3	0.395 ± 0.098	142 ± 5 (98%)
**PLGA-DIVEMA-N**	0	165 ± 2	0.290 ± 0.050	175 ± 3 (100%)
	0.5	160 ± 1	0.300 ± 0.080	176 ± 3 (100%)
	1	160 ± 1	0.300 ± 0.078	170 ± 2 (100%)
	2	350 ± 3	0.425 ± 0.102	380 ± 5 (92%)
	4	382 ± 3	0.398 ± 0.062	310 ± 6 (96%)
	6	379 ± 3	0.496 ± 0.005	390 ± 5 (90%)
**PLGA without shell**	0	87 ± 1	0.153 ± 0.003	85 ± 1 (100%)
	0.5	88 ± 1	0.153 ± 0.008	79 ± 1 (100%)
	1	90 ± 2	0.179 ± 0.025	78 ± 4 (100%)
	2	94 ± 3	0.184 ± 0.042	83 ± 1 (100%)
	4	89 ± 5	0.148 ± 0.002	77 ± 9 (100%)
	6	104 ± 9	0.241 ± 0.016	90 ± 9 (97%)

Table S2. The size and size distribution of core-shell nanoparticles upon incubation in PBS. Mean ± SD, n=3.

Nanoparticle type	Time, h	Nanoparticle size and size distribution		
		Mean diameter. nm	PDI	Volume size distribution, nm
**PLGA/HSA-C**	0	159 ± 4	0.302 ± 0.010	134 ± 5 (100%)
	0.5	152 ± 3	0.257 ± 0.018	132 ± 4 (98%)
	1	145 ± 3	0.218 ± 0.010	132 ± 4 (96%)
	2	139 ± 3	0.270 ± 0.010	137 ± 5 (94%)
	4	152 ± 6	0.236 ± 0.001	130 ± 7 (95%)
	6	150 ± 4	0.248 ± 0.013	133 ± 5 (94%)
**PLGA/HSA-A cross-linked**	0	148 ± 2	0.185 ± 0.002	140 ± 4 (93%)
	0.5	146 ± 1	0.186 ± 0.018	138 ± 2 (100%)
	1	146 ± 2	0.186 ± 0.016	152 ± 3 (100%)
	2	147 ± 1	0.207 ± 0.001	136 ± 2 (100%)
	4	151 ± 1	0.234 ± 0.002	137 ± 3 (100%)
	6	151 ± 3	0.227 ± 0.034	141 ± 5 (100%)
**PLGA/HSA-A non-cross-linked**	0	136 ± 1	0.102 ± 0.011	135 ± 3 (100%)
	0.5	133 ± 1	0.122 ± 0.040	135 ± 2 (100%)
	1	134 ± 1	0.105 ± 0.050	131 ± 2 (100%)
	2	136 ± 1	0.104 ± 0.003	134 ± 2 (100%)
	4	140 ± 2	0.167 ± 0.006	133 ± 4 (100%)
	6	135 ± 1	0.109 ± 0.001	133 ± 2 (100%)
**PLGA/HSA-IE cross-linked**	0	105 ± 2	0.127 ± 0.012	95 ± 3 (100%)
	0.5	95 ± 3	0.089 ± 0.021	84 ± 5 (100%)
	1	93 ± 3	0.089 ± 0.025	83 ± 5 (100%)
	2	98 ± 1	0.079 ± 0.011	91 ± 3 (100%)
	4	97 ± 2	0.105 ± 0.007	87 ± 5 (100%)
	6	107 ± 45	0.205 ± 0.009	90 ± 30 (96%)
**PLGA/HSA-IE non–cross-linked**	0	96 ± 2	0.071 ± 0.001	85 ± 5 (100%)
	0.5	90 ± 1	0.079 ± 0.019	81 ± 3 (100%)
	1	89 ± 1	0.071 ± 0.021	82 ± 3 (100%)
	2	91 ± 1	0.069 ± 0.008	82 ± 2 (100%)
	4	90 ± 1	0.105 ± 0.001	87 ± 3 (100%)
	6	92 ± 1	0.126 ± 0.007	82 ± 3 (100%)
**PLGA-DIVEMA-N**	0	218 ± 3	0.179 ± 0.015	230 ± 5 (96%)
	0.5	202 ± 4	0.148 ± 0.008	218 ± 7 (98%)
	1	200 ± 3	0.155 ± 0.004	227 ± 6 (100%)
	2	194 ± 4	0.147 ± 0.045	213 ± 7 (100%)
	4	182 ± 4	0.139 ± 0.020	191 ± 7 (100%)
	6	175 ± 3	0.124 ± 0,017	188 ± 5 (100%)
**PLGA without shell**	0	110 ± 3	0.085 ± 0.010	108 ± 2 (100%)
	0.5	113 ± 2	0.090 ± 0.011	105 ± 3 (100%)
	1	114 ± 3	0.092 ± 0.020	110 ± 1 (100%)
	2	116 ± 4	0.121 ± 0.016	104 ± 8 (100%)
	4	118 ± 4	0.114 ± 0.045	111 ± 5 (100%)
	6	119 ± 3	0.160 ± 0.055	105 ± 8 (100%)

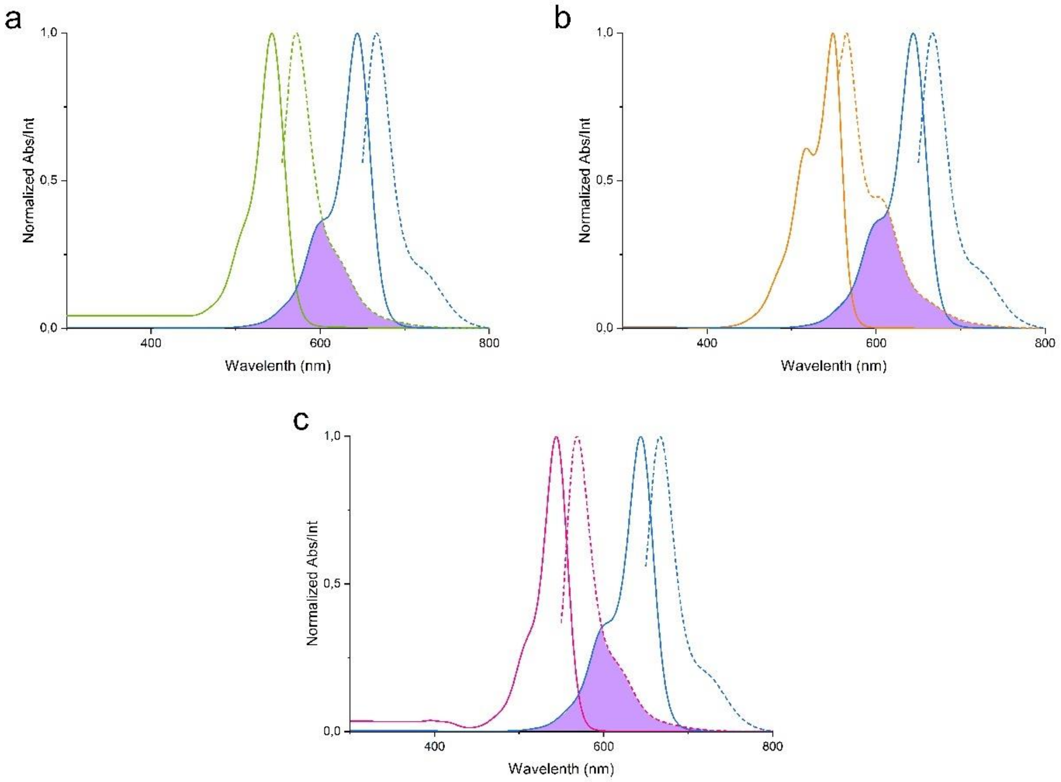

Figure S7. Normalized spectra of potential FRET pairs using Cy5 as the acceptor. (a) Rhodamine B isothiocyanate (RhBITC, green line)—Cy5 (blue line), (b) Cy3 (orange line)—Cy5 (blue line), and (c) Rhodamine B (RhB, purple line)—Cy5 (blue line). Absorption spectra (EtOH, 1 µg/mL) were recorded with a UV-1900i spectrophotometer (Shimadzu, Japan) over a wavelength range of 300–800 nm, while fluorescence spectra (EtOH, 1 µg/mL) were acquired using an RF-6000 fluorimeter (Shimadzu, Japan). The excitation wavelengths were 550 nm for RhBITC and Cy3, 545 nm for RhB, and 645 nm for Cy5. Spectra were normalized (0–1) using OriginLab Pro software. Absorbance spectra are depicted as solid lines, and fluorescence spectra as dashed lines. The overlap between the donor emission spectrum and the acceptor absorption spectrum indicates FRET efficiency.

Table S3. Fluorescent properties of free dyes. Representative data.

Fluorescent dyes	ε, M^{-1} cm^{-1} (λ_{ex}, nm; solvent)	Quantum Yield	Brightness, M^{-1} cm^{-1}
RhB	106,000 (545, ethanol)	0.7	$7.42x10^4$
Cy5	250,000 (644, ethanol)	0.20	$5.00x10^4$
Cy3	150,000 (555, ethanol)	0.31	$4.65x10^4$
RhBITC	106,000 (554, ethanol)	0.49	$5.19x10^4$

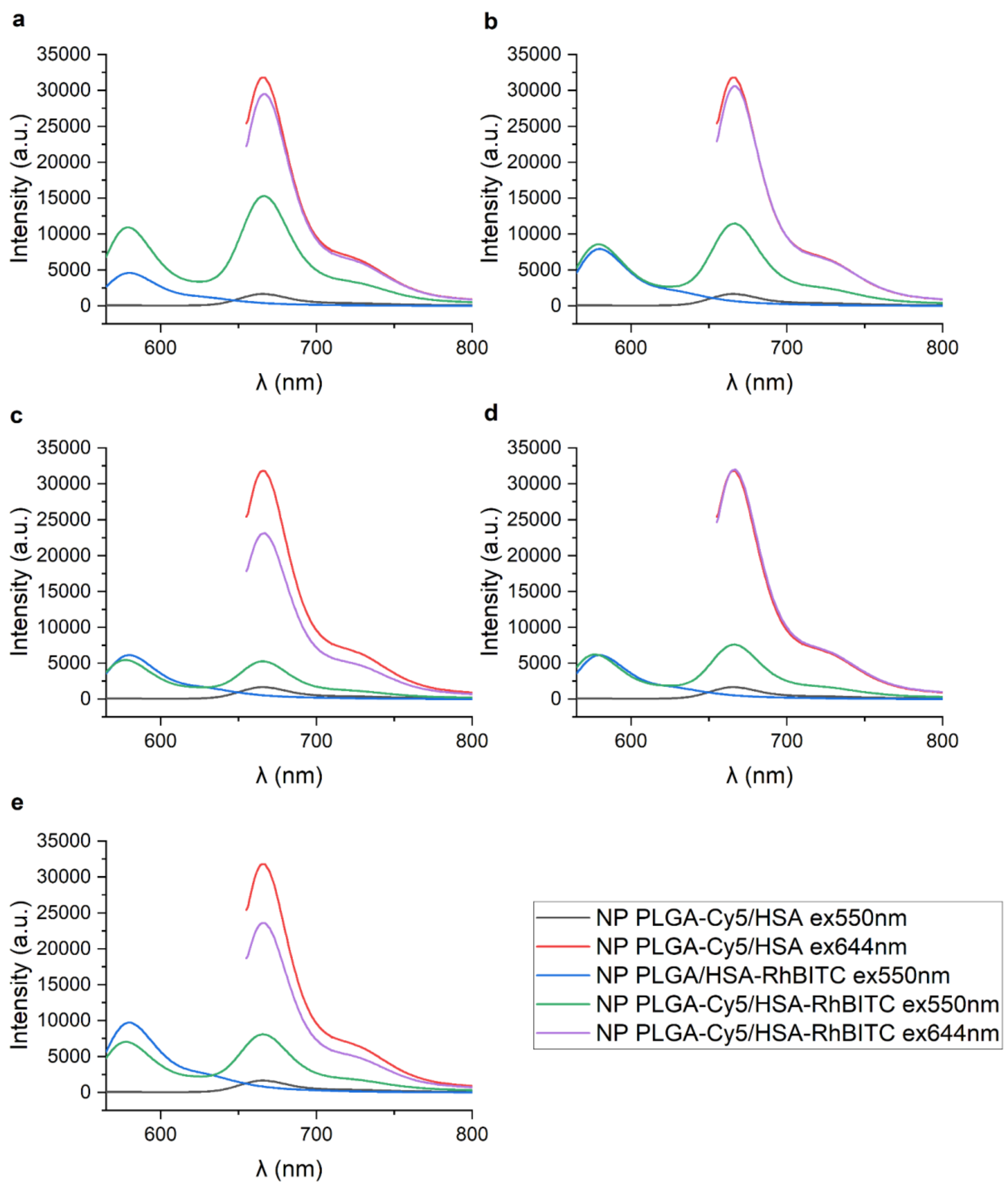

Figure S8. Fluorescent spectra of PLGA-Cy5/HSA-RhBITC NP in water. (a) PLGA/HSA-IE non-cross-linked; (b) PLGA/HSA-IE cross-linked; (c) PLGA/HSA-A non-cross-linked; (d) PLGA/HSA-A cross-linked; (e) PLGA/HSA-C.

Table S4. Estimation of the percentage of shell remaining on the nanoparticle surface after 6 hours of incubation in PBS in the presence and absence of 4.5% HSA, pH = 7.4. Representative data.

Nanoparticle type	Percent of the shell remaining on NP surface					
	PBS			PBS + 4.5% HSA		
	Time, h					
	0	1	6	0	1	6
**PLGA/HSA-C**	68.8	67.7	54.7	42.7	32.5	15.7
**PLGA/HSA-A cross-linked**	84.9	84.0	70.8	53.4	42.7	23.3
**PLGA/HSA-A non-cross-linked**	66.0	62.3	50.3	40.9	31,7	10.7
**PLGA/HSA-IE cross-linked**	86.9	86.8	75.4	38.4	32.9	22.7
**PLGA/HSA-IE non–cross-linked**	80.3	77.5	69.9	23.2	20.5	7.8
**PLGA-DIVEMA-N**	52.1	52.1	50.0	37.6	29.9	28.6

Table S5. Estimation of the percentage of shell remaining on the nanoparticle surface after 6 hours of incubation in PBS in the presence of 4.5% HSA, pH = 5.5. Representative data.

Nanoparticle type	Percent of the shell remaining on NP surface					
	Time, h					
	0	0.5	1	2	4	6
**PLGA/HSA-C**	39.9	34.6	24.4	20.9	20.9	17.2
**PLGA/HSA-A cross-linked**	43.3	42.5	39.4	29.3	27.3	21.6
**PLGA/HSA-A non-cross-linked**	36	31.2	20.8	19.4	14.2	9.4
**PLGA/HSA-IE cross-linked**	36.6	25.8	18.4	16.1	16.1	15.7
**PLGA/HSA-IE non–cross-linked**	27.5	25.7	22.9	7.3	6	5.2
**PLGA-DIVEMA-N**	31.3	29.4	29.4	27.7	27.6	27.2

Table S6. Colocalization between the **PLGA-Cy5 core** and **HSA-RhBITC shell** and **lysosomes** in Gl261 cells. Investigation of the core/shell structure integrity over time. (incubation times: 15, 30, 45 min; mean ± SD, n=3).

Incubation time, min	**Manders' overlap coefficient**				
	Between PLGA-Cy5 and HSA-RhBITC core/shell integrity				
Sample	**PLGA/HSA-C**	**PLGA/HSA-IE cross-linked**	**PLGA/HSA-IE non-cross-linked**	**PLGA/HSA-A cross-linked**	**PLGA/HSA-A non-cross-linked**
15	0.8372 ± 0.0683	0.6302 ± 0.1363	0.6050 ± 0.0871	0.8728 ± 0.0412	0.8423 ± 0.0347
30	0.8761 ± 0.04508	0.6567 ± 0.1204	0.6338 ± 0.0911	0.9152 ± 0.0219	0.8778 ± 0.0437
45	0.8897 ± 0.0304	0.7344 ± 0.0557	0.7098 ± 0.0352	0.9234 ± 0.0155	0.8810 ± 0.0967
	Between PLGA-Cy5 (core) and lysosomes				
15	0.3588 ± 0.0610	0.4448 ± 0.1091	0.3926 ± 0.0949	0.3909 ± 0.0828	0.4189 ± 0.0429
30	0.4018 ± 0.0851	0.4509 ± 0.0855	0.4052 ± 0.0717	0.3897 ± 0.0641	0.4643 ± 0.1133
45	0.4065 ± 0.0612	0.5353 ± 0.0934	0.4166 ± 0.0509	0.4124 ± 0.0663	0.3400 ± 0.0427
	Between HSA-RhBITC and lysosomes				
15	0.5772 ± 0.0513	0.7844 ± 0.0791	0.7367 ± 0.0466	0.4520 ± 0.0759	0.5833 ± 0.0640
30	0.5993 ± 0.0873	0.7884 ± 0.0484	0.7082 ± 0.0803	0.4435 ± 0.0637	0.5934 ± 0.1266
45	0.6088 ± 0.0690	0.8318 ± 0.0341	0.6929 ± 0.0488	0.4358 ± 0.0654	0.3684 ± 0.0914

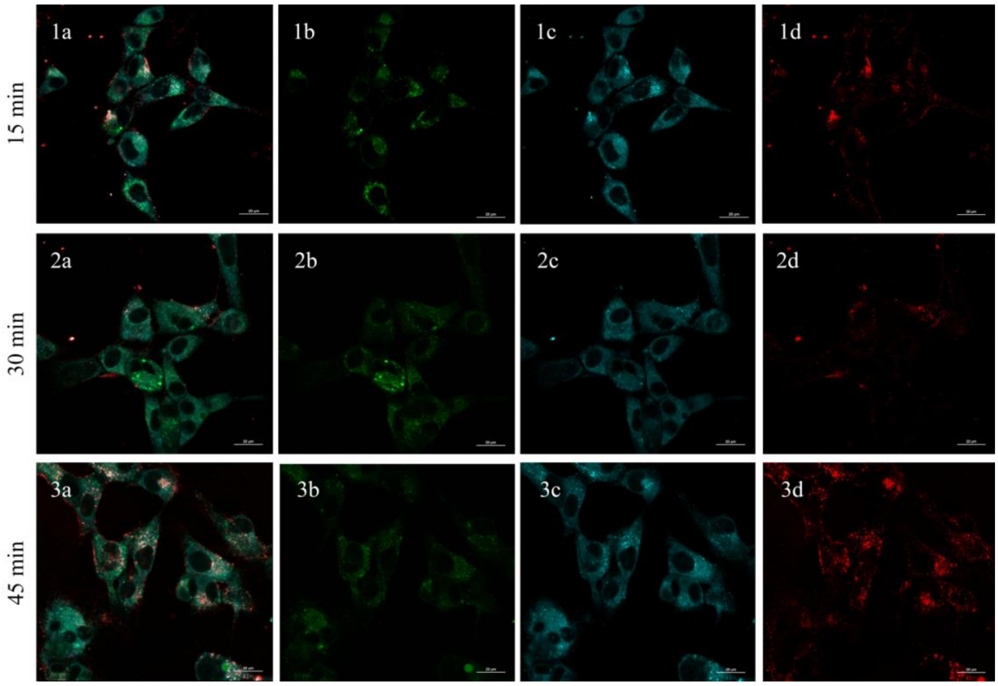

Figure S9. Confocal images of Gl261 cells after incubation with PLGA/HSA-IE cross-linked NP. (a) merged images; (b) lysosomes (green, LysoTracker® Green DND-26); (c) shell (cyan, HSA-RhBITC); (d) core (red, PLGA-Cy5). CSLM. Scale bar: 20 µm.

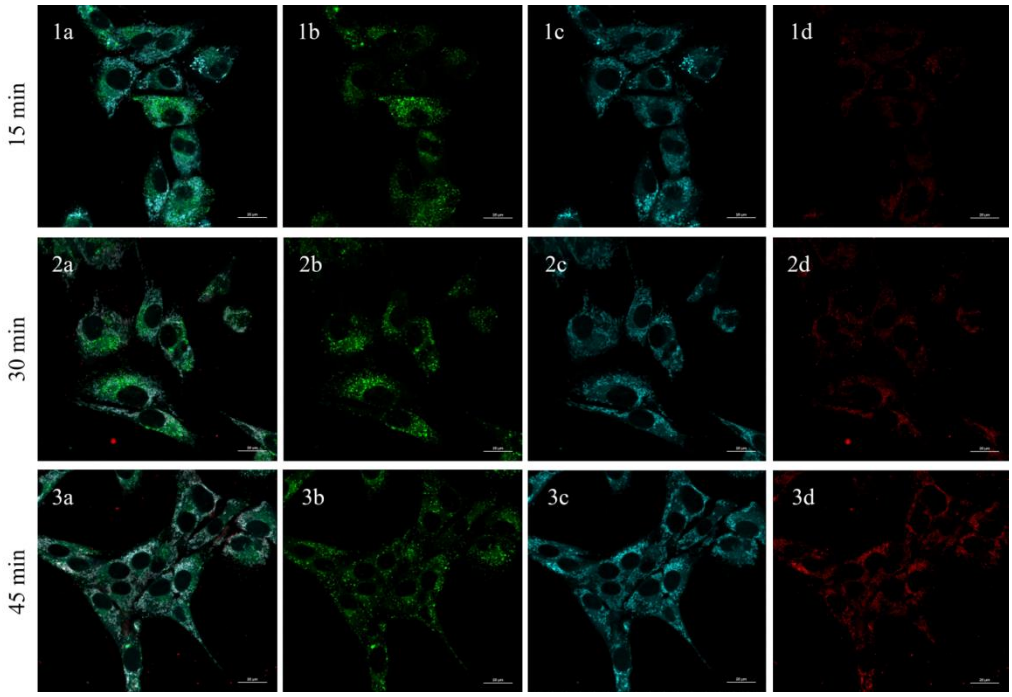

Figure S10. Confocal images of Gl261 cells after incubation with PLGA/HSA-A-C NP. (a) merged images; (b) lysosomes (green, LysoTracker® Green DND-26); (c) shell (cyan, HSA-RhBITC); (d) core (red, PLGA-Cy5). CSLM. Scale bar: 20 μm.

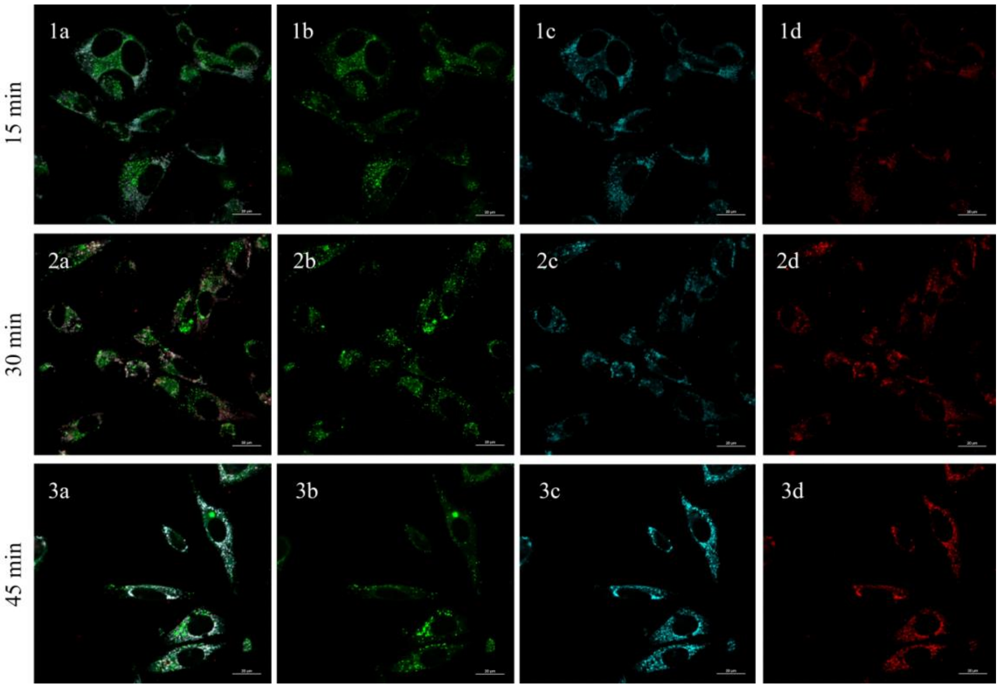

Figure S11. Confocal images of Gl261 cells after incubation with PLGA/HSA-A cross-linked NP. (a) merged images; (b) lysosomes (green, LysoTracker® Green DND-26); (c) shell (cyan, HSA-RhBITC); (d) core (red, PLGA-Cy5). CSLM. Scale bar: 20 μm.

Table S7. Colocalization between the **PLGA-Cy5 core** and **DIVEMA-Cy3 shell** and **lysosomes** in Gl261 cells. Investigation of the core/shell structure integrity over time (incubation times: 15, 30, 45 min; mean ± SD, n=3).

Incubation time, min	**Manders' overlap coefficients**
	Between PLGA-Cy5 and DIVEMA-Cy3 core/shell integrity
15	0.6340 ± 0.0822
30	0.7916 ± 0.0277
45	0.7696 ± 0.0550
	Between PLGA-Cy5 (core) and lysosomes
15	0.3959 ± 0.0857
30	0.4966 ± 0.04216
45	0.4668 ± 0.0673
	Between Cy3 and lysosomes
15	0.4969 ± 0.0917
30	0.5621 ± 0.0418
45	0.5704 ± 0.0872

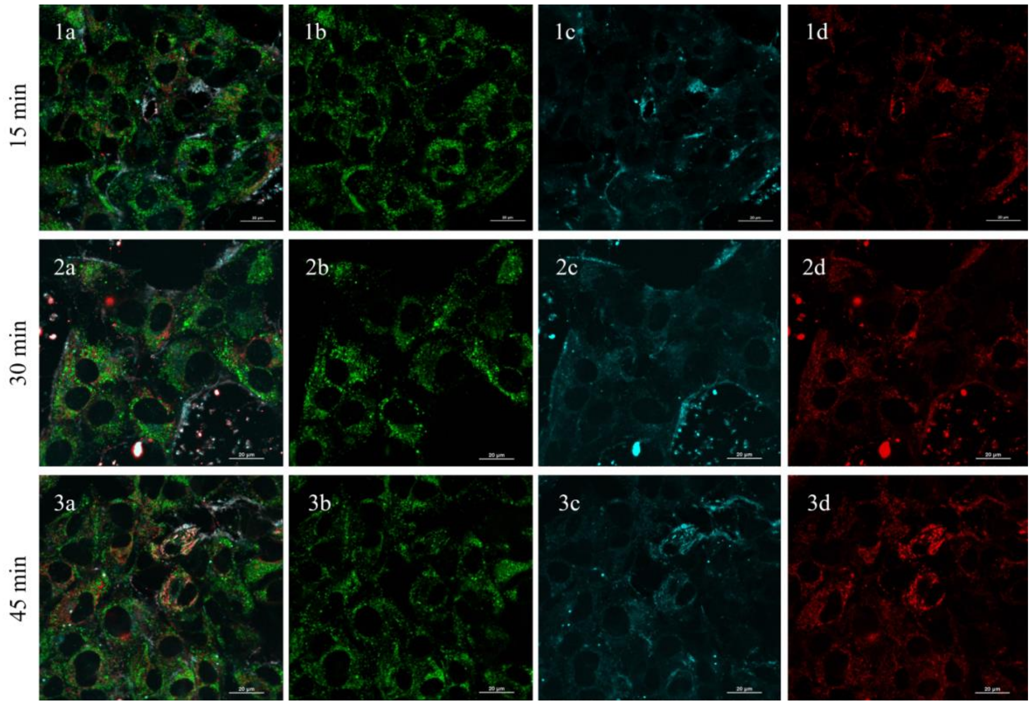

Figure S12. Confocal images of Gl261 cells after incubation with PLGA/DIVEMA-N NP. (a) merged images; (b) lysosomes (green, LysoTracker® Green DND-26); (c) shell (cyan, DIVEMA-Cy3); (d) core (red, PLGA-Cy5). CSLM. Scale bar: 20 µm.

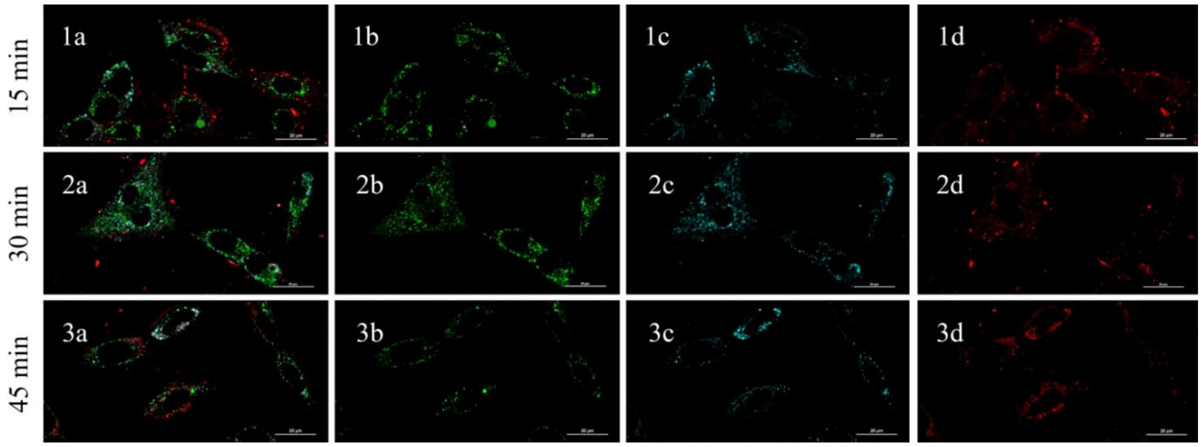

Figure S13. Confocal images of Gl261 cells after incubation with PLGA/P188 NP. (a) merged images; (b) lysosomes (green, LysoTracker® Green DND-26); (c) shell (P188-RhB); (d) core (red, PLGA-Cy5). CSLM. Scale bar: 20 μm.

Table S8. Colocalization between the **PLGA-Cy5 core** and **P188-RhB shell** and **lysosomes** in Gl261 cells. Investigation of the core/shell structure integrity over time (incubation times: 15, 30, 45 min; mean ± SD, n=3).

Incubation time, min	**Manders' overlap coefficients**
	Between PLGA-Cy5 and P188-RhB core/shell integrity
15	0.4981 ± 0.0494
30	0.6085 ± 0.0951
45	0.6241 ± 0.1325
	Between PLGA-Cy5 (core) and lysosomes
15	0.2810 ± 0.0791
30	0.3243 ± 0.0684
45	0.2774 ± 0.0829
	Between P188-RhB and lysosomes
15	0.3423 ± 0.0464
30	0.3895 ± 0.0755
45	0.4173 ± 0.0350

Table S9. Evaluation of **PLGA-Cy5/HSA-RhBITC NP** uptake by Gl261 cells at different time points. The data are represented as percentages of Cy5-positive and double-positive cells (mean ± SD, n=3).

Sample	Percent of positive cells									
	PLGA/HSA-C		PLGA/HSA-A cross-linked		PLGA/HSA-A non-cross-linked		PLGA/HSA-IE cross-linked		PLGA/HSA-IE non-cross-linked	
Incubation time, min	Cy5-positive	Double positive	Cy5-positive	Double positive	Cy5-positive	Double positive	Cy5-positive	Double positive	Cy5-positive	Double positive
15	26.79 ± 5.43	20.32 ± 4.66	26.93 ± 3.66	6.89 ± 1.51	32.12 ± 2.32	14.80 ± 1.85	65.29 ± 2.66	6.73 ± 1.59	63.62 ± 2.189	6.79 ± 1.47
30	52.78 ± 2.58	45.16 ±2.86	48.50 ± 4.63	17.49 ± 4.49	55.42 ± 3.92	31.50 ± 7.17	78.94 ± 4.38	14.50 ± 1.45	74.22 ± 7.04	17.28 ± 4.65
45	81.10 ± 4.48	76.69 ± 4.94	68.88 ± 3.16	31.52 ± 1.37	71.51 ± 8.77	43.46 ± 2.11	89.11±1.02	32.27 ± 1.24	88.01 ± 2.61	42.06 ± 5.27

Table S10. Evaluation of **PLGA-Cy5/P188-RhB** uptake by Gl261 cells at different time points. The data are represented as percentages of Cy5-positive and double-positive cells (mean ± SD, n=3).

Incubation time, min	Cy5-positive (%)	Double positive (%)
15	59.19 ± 7.79	14.79 ± 1.85
30	87.70 ± 3.38	31.50 ± 7.17
45	91.02 ± 3.14	43.46 ± 2.11

Table S11. Evaluation of **PLGA-Cy5/DIVEMA-Cy3** NP uptake by Gl261 cells at different time points. The data are represented as percentages of Cy5-positive and double-positive cells (mean ± SD, n=3).

Incubation time, min	Cy5-positive (%)	Double positive (%)
15	49.05 ± 2.28	10.55 ± 2.36
30	69.72 ± 4.37	10.03 ± 0.82
45	83.38 ± 2.45	9.17 ± 1.90
